# Dnmt3a is downregulated by Stat5a and mediates G0/G1 arrest by suppressing the miR-17-5p/Cdkn1a axis in Jak2^V617F^ cells

**DOI:** 10.1186/s12885-021-08915-0

**Published:** 2021-11-13

**Authors:** Jie Zhou, Cheng Guo, Hao Wu, Bing Li, Li-Li Zhou, Ai-Bin Liang, Jian-Fei Fu

**Affiliations:** 1 Tongji University School of Medicine, Shanghai, 200092 China; 2 Department of Gastroenterology, Tongji Hospital of Tongji University, Shanghai, 200065 China; 3 Department of Hematology, Tongji Hospital of Tongji University, Tongji University School of Medicine, No.389 Xincun Road, Putuo District, Shanghai, 200065 China

**Keywords:** Classical myeloproliferative neoplasms, Jak2^V617F^, Stat5a, Dnmt3a, Cdkn1a, GAS

## Abstract

**Background:**

Despite of the frequently reported Dnmt3a abormality in classical myeloproliferative neoplasms (cMPNs) patients, few research explores how the Dnmt3a is regulated by Jak2^V617F^ mutation. In this study, we have investigated how the Dnmt3a is regulated by Jak2^V617F^ mutation and its effects on downstream signaling pathways in cMPNs.

**Methods:**

Specimens of Jak2^V617F^ positive cMPN patients and normal controls were collected. Murine BaF3 cell line was used to construct cell models. Dual-Glo luciferase assays and chromatin immunoprecipitation (ChIP)-qPCR were performed to detect the impact of Stat5a on transcription activity of Dnmt3a. Soft agar colony formation assay and cell counting assay were performed to detect cell proliferation. BrdU staining and flow cytometry were used to investigate cell cycle distribution. Western blotting and quantitative reverse-transcription PCR (qPCR) were performed to detect the expression levels of genes.

**Results:**

Firstly, the results of western blotting and qPCR revealed that compared with the control samples, Dnmt3a is downregulated in Jak2^V617F^ positive samples. Then we explored the mechanism behind it and found that Dnmt3a is a downstream target of Stat5a, the transcription and translation of Dnmt3a is suppressed by the binding of aberrantly activated Stat5a with Dnmt3a promoter in Jak2^V617F^ positive samples. We further revealed the region approximately 800 bp upstream of the first exon of the Dnmt3a promoter, which includes a gamma-activated sequence (GAS) motif of Stat5a, is the specific site that Stat5a binds to. Soft agar colony formation assay, cell counting assay, and BrdU staining and flow cytometry assay found that Dnmt3a in Jak2^V617F^-BaF3 cells significantly affected the cell proliferation capacity and cell cycle distribution by suppressing Cdkn1a via miR-17-5p/Cdkn1a axis and mediated G0/G1 arrest.

**Conclusions:**

Transcription and translation of Dnmt3a is downregulated by the binding of Stat5a with Dnmt3a promoter in Jak2^V617F^ cells. The GAS motif at promoter of Dnmt3a is the exact site where the Stat5a binds to. Dnmt3a conducted G0/G1 arrest through regulating miR-17-5p/Cdkn1a axis. The axis of Stat5a/Dnmt3a/miR-17-5p/Cdkn1a potentially provides a treatment target for cMPNs.

**Supplementary Information:**

The online version contains supplementary material available at 10.1186/s12885-021-08915-0.

## Background

Classical myeloproliferative neoplasms (cMPNs) are characterized by the overproduction of terminally differentiated blood cells [1], including polycythemia vera (PV), essential thrombocythemia (ET) and primary myelofibrosis (PMF) [2]. The acquired somatic mutation of the tyrosine kinase JAK2 gene (JAK2^V617F^) is the most important pathogenesis of cMPNs [3]. DNA methyltransferase (DNMT) 3a is a de novo methyltransferase [4] which acts as a haplotype tumor suppressor gene in myeloid leukemia. Previous researches have reported the frequent incidence of DNMT3a mutation and disregulation in myeloid leukemia and cMPNs patients [5–7]. Jacquelin et al. has reported that there is a synergistic carcinogenic effect in cMPNs driven by JAK2^V617F^ mutation and DNMT3a deletion, which leads to the activation of inflammatory signals driven by hematopoietic stem and progenitor cell (HSPC) enhancers [6].

These above studies indicated that as well as the JAK2 gene, the DNMT3a gene is also important in the pathogenesis of cMPNs, but their relationship in cMPNs is still a mystery. Few research has answered the question how the Dnmt3a is regulated by Jak2^V617F^ mutation, and its effects on downstream signaling pathways in cMPNs still needs further research.

In order to answer the question, we carried out the present study. Firstly, we found that Dnmt3a was downregulated at the transcriptional and translational levels in Jak2^V617F^-positive BaF3 cells (hereinafter referred to as Jak2^V617F^ BaF3). Further research showed that the binding of Stat5a protein (which was abnormal activated by the Jak2^V617F^ mutation) with Dnmt3a promoter is the mechanism of this downregulation. The gamma-activated sequence (GAS) motif of Stat5a at the region approximately 800 bp upstream of the first exon of the Dnmt3a promoter is the specific site of the binding. Dnmt3a significantly affected the cell proliferation capacity and cell cycle distribution and mediated G0/G1 arrest by suppressing the Cdkn1a via miR-17-5p in Jak2^V617F^ BaF3 cells. This Stat5a/Dnmt3a/miR-17-5p/Cdkn1a regulation axis identified in this study might play an important role in cMPNs and might serve as a potential treatment target for cMPNs.

## Methods

### Patients and samples

A total of 12 samples (six JAK2^V617F^-positive cMPNs patients and six normal controls) were recruited from Tongji hospital of Tongji University (Table S1, shown in Additional file 1). The diagnosis of cMPNs was defined according to World Health Organization (WHO) criteria [8]. The use of clinical samples in our study was according to the Declaration of Helsinki and was approved by the Medical ethic committee of Tongji hospital of Tongji University on Feb. 2021 (Number: 2021-KYSB-177). Written informed consent was obtained from each participant. Mononuclear cells are isolated from bone marrow using density gradient separation (Percoll, Solarbio life sciences, Beijing, China). Total RNA was extracted from bone marrow mononuclear cells using Trizol reagent (Invitrogen, Carlsbad, CA, USA) following the manufacturer’s instruction. Total RNA from each sample was quantified by the NanoDrop ND-1000 and RNA integrity was assessed by standard denaturing agarose gel electrophoresis.

### Cell lines and cell culture

The human erythroleukemia cell line HEL, human immortalised myelogenous leukemia cell line K562, human caucasian bone marrow acute myelogenous leukaemia cell line KG1α, human myeloid leukaemia cell line U937, human myeloid leukaemia cell line NB4, human myeloid leukaemia cell line THP1, the murine pro B cell line BaF3 and 293 T/17 cells were purchased from the Cell Bank, Chinese Academy of Sciences (Shanghai, China) and were characterized using Short Tandem Repeat (STR) markers. HEL, U937, NB4, THP1 and BaF3 cells were grown in Roswell Park Memorial Institute (RPMI) 1640 Medium (Gibco; Thermo Fisher Scientific, Inc.), and wild-type BaF3 cells were grown with 1 ng/mL IL-3. 293 T/17 cells were grown in Dulbecco’s modified Eagle’s medium (Gibco; Thermo Fisher Scientific, Inc.). K562 and KG1α cells were grown in Iscove’s Modified Dulbecco’s Medium (IMDM, Gibco; Thermo Fisher Scientific, Inc.). All media were supplemented with 1% penicillin-streptomycin (Gibco; Thermo Fisher Scientific, Inc.) and 10% fetal bovine serum (FBS; Gibco; Thermo Fisher Scientific, Inc.). All cell lines were cultured in a humidified atmosphere of 5% CO_2_ at 37 °C and were found to be negative for mycoplasma contamination.

### Plasmid construction and cell transfection

Cytokine-dependent wild type BaF3 cells were transformed to Jak2^V617F^ BaF3 cells with growth factor independence by ectopic expression of Jak2^V617F^, resulting in constitutive phosphorylation of Jak2 as well as downstream targets, such as Stat5a. The ectopic expression plasmid of Jak2^V617F^ was generated by inserting the full-length Jak2 with Jak2V617F mutation, which was generated by site-directed mutagenesis and confirmed by sequencing, into the pLVX-zsGreen vector (Clontech Laboratories, Inc.). The wild type BaF3 cells transformed empty vector were used as control. The Stat5a overexpression plasmid was generated by inserting the Stat5a coding DNA sequence (CDS) into the pLVX-Puro vector. The Dnmt3a overexpression plasmid was generated by inserting the Dnmt3a CDS into the pLVX-zsGreen vector (Clontech Laboratories, Inc.). shRNA lentiviral plasmids targeting Stat5a, Dnmt3a and Cdkn1a were constructed by inserting annealed shRNA template DNA sequences into pLVX-shRNA (Clontech Laboratories, Inc.) vector containing either a puromycin resistance cassette or ZsGreen, respectively. The effective targeting shRNA sequences of the Stat5a gene were 5′-GCCAGATGCAAGTGTTGTA-3′ and 5′-GCACCTTCAGATCAACCAA-3′. The effective targeting shRNA sequences of the Dnmt3a gene were 5′-CCAGATGTTCTTTGCCAATAA-3′ and 5′-GCAGACCAACATCGAATCCAT-3′. The effective targeting shRNA sequences of the Cdkn1a gene were 5′-CCGAGAACGGTGGAACTTT-3′ and 5′-GCAAAGTGTGCCGTTGTCT-3′. The sequence 5′-GCGCGCTTTGTAGGATTCG-3′, which is unrelated to any sequence in humans and mice, served as a negative control (shRNA-Ctrl). To produce lentiviral particles, all of the aforementioned plasmids (with two packaging plasmids, pCMV-dR8.2 dvpr and pCMV-VSV-G, at a mass ratio of 4:3:2) were cotransfected into 293 T/17 cells via the calcium phosphate precipitation method (CPT high-efficiency transfection kit, Wuhan Viraltherapy Technologies Co. Ltd) according to the manufacturer’s instructions. Cell transfection with lentivirus supernatant was performed with 10 μg/ml polybrene, and GFP or puromycin resistance expression in cells was tested after 48–72 h. As a high level of Stat5a depletion would result in significant inhibition of cell growth and massive cell death, we repeated the construction of stable-transfected cells for each assay. For luciferase reporter plasmids, different promoter regions of Dnmt3a (− 2860 bp to + 140 bp, Table S2, shown in Additional file 2) were cloned into the pmirGLO dual-luciferase expression vector (Promega (Beijing) Biotech Co. Ltd). Luciferase reporter plasmids were transfected into 293 T/17 cells via the calcium phosphate precipitation method. RNAFit reagent was used for transfection of miRNAs following the manufacturer’s protocol (Hanbio Technology (Shanghai) Co. Ltd).

### Reagents, chemicals, and antibodies

Stat5a inhibitor AZ960 (cat. no. S2214) and LY2784544 (cat. no. S2179) were obtained from Selleckchem (USA) and dissolved in DMSO (Sigma-Aldrich; Merck KGaA). Primary antibodies against Jak2 (cat. no. 3230), p-Jak2 (cat. no. 3771), Stat5a (cat. no. 94205), p-Stat5a (cat. no. 4322), Dnmt3a (cat. no. 3598), β-actin (cat. no. 8457) and Cdkn1a (cat. no. 64016) were purchased from Cell Signaling Technology (USA). Mmu-miR-17-5p (hereinafter referred to as miR-17-5p) mimic, related mimics control, inhibitor and related inhibitor control were got from GenePharma (China). The sequences of miRNA were listed in Table 1.

**Table 1:** Sequences of miRNA mimic, related mimics control, inhibitor and related inhibitor control

	Sense 5′-3′	Antisense 5′-3′
miR-17-5p mimic	CAAAGUGCUUACAGUGCAGGUAG	ACCUGCACUGUAAGCACUUUGUU
miR-17-5p inhibitor	CUACCUGCACUGUAAGCACUUUG	/
Negative control (NC)	UUCUCCGAACGUGUCACGUTT	ACGUGACACGUUCGGAGAATT
Inhibitor NC (NC in)	CAGUACUUUUGUGUAGUACAA	/

### Western blotting

Total protein was extracted with RIPA buffer (Epizyme Biotech, cat. no. P1101) containing protease inhibitor cocktail (Beyotime Biotechnology, cat. The BCA method (Beyotime Biotechnology, cat.no. P0011) was used to detect the protein concentration. Equal amounts of total protein (30 μg) were separated by protein electrophoresis on a 10% SDS-PAGE gel and transferred onto PVDF membranes (Millipore, Billerica, MA). The membranes were probed with the indicated antibodies. Protein bands were detected and quantified by Amersham Imager 600 (GE Healthcare).

### Dual-luciferase reporter assay

293 T/17 cells were co-transfected with different luciferase reporter plasmids and a Stat5a overexpression plasmid for 48 h for the luciferase reporter assay of the Dnmt3a promoter. Luciferase activities were measured with the Dual Luciferase Reporter Gene Assay Kit (Beyotime Biotechnology, cat.no. RG027). Data were normalized by calculating the ratio between firefly and Renilla luciferase activity.

### Cell counting assay for proliferation detection

Cells in good growth conditions to be tested were centrifuged, the supernatant was removed, new medium was added, and the cells were resuspended by gentle blowing, and cell counting was performed. Fifty cells were inoculated in each well of a 24-well plate and incubated in a humidified atmosphere of 5% CO_2_ at 37 °C. A total of 5 groups of 3 wells each were counted. The cells were counted in one group every three days from the 4th day of incubation. The cell growth curve was plotted as a line graph based on the count of each group of cells.

### Soft agarose colony formation assay

The 1% agarose solution was prepared with ultrapure water, autoclaved at 121 °C for 15 min, placed in a water bath at 42 °C, mixed with 2× complete medium containing 20% FBS in equal volumes to make the final concentration of agarose 0.5%, and poured rapidly into 6-well cell culture plates at 4 mL per well. Equilibrate the 6-well cell culture plates in a 37 °C incubator for more than 30 min before use. The prepared 0.7% agarose solution was placed in a 42 °C-water bath. The cells were resuspended in single-cell suspensions with culture medium, counted, and diluted to 1 × 10^3^ cells/mL (100 cells per well). Then, 0.2 mL of the cytosol, 2 mL of 2× cell culture medium containing 20% FBS and 2 mL of 0.7% agarose were added to each tube, mixed well and spread on the bottom agar layer (2 mL per well, with 3 replicate wells in total). After solidification at room temperature, the 6-well cell culture plates were incubated at 37 °C for 14 d. Cells were observed under a microscope after continuous culture for 14 d. The number of colonies with more than 10 cells was counted.

### Cell cycle distribution analysis

For cell cycle distribution analysis, BrdU solution was added to the medium at a final concentration of 10 ng/ml. After culturing at 37 °C for 2 h, the cells were collected, washed twice with PBS and fixed with 95% ethanol overnight at 4 °C. The next day, the cells were washed twice with PBS buffer (1% FBS, 0.09% NaN3). The cells were resuspended in 2 ml of PBS buffer (2 N HCl and 0.5% Triton X-100) and incubated in the dark for 30 min at room temperature. The cells were washed once with 1 ml of PBS and resuspended in 0.5 ml of 0.1 M Na_2_B_4_O_7_ for 2 min. The cells were washed again and resuspended in 50 μl of the anti-BrdU APC antibody solution (diluted with staining buffer at 1:20; cat. no. ab136650, Abcam) and incubated for 30–60 min. Then, 5 μl of PI staining solution was added and incubated for 10 min. The cell cycle distribution was determined by a flow cytometer (BD Biosciences) and analyzed by FlowJo V10.

### RNA extraction and qPCR

Total RNA was extracted with a Quick-RNA MicroPrep RNA Extraction Kit (Zymo Research, USA), cDNA of coding genes was produced by PrimeScript RT Master Mix (Takara, Japan), and cDNA of miRNAs was produced by TransScript miRNA First-Strand cDNA Synthesis SuperMix (Transgene, China). The ChamQ Universal SYBR qPCR Master Mix Kit (Vazyme, China) was used to detect relative mRNA expression by a LightCycler 96 system (Roche, Switzerland) with 40 cycles of PCR thermocycling. The primer sequences were as follows: For BaF3 cells: β-actin forward, 5′-GTGACGTTGACATCCGTAAAGA-3′ and reverse, 5′-GCCGGACTCATCGTA CTCC-3′; Dnmt3a forward, 5′-GATGAGCCTGAGTATGAGGATGG-3′ and reverse, 5′-CAAGA CACAATTCGGCCTGG-3′; Stat5a forward, 5′-CAGATGCAAGTGTTGTATGGGC-3′ and reverse, 5′-GCTGGCTCTCGATCCACTG-3′; Cdkn1a forward, 5′-CCTGGTGATGTCCGAC CTG-3′ and reverse, 5′-CCATGAGCGCATCGCAATC-3′. For specimens of cMPN patients and normal controls: DNMT3a forward, 5′-GGAGGACCGAAAGGACGGA-3′ and reverse, 5′-CCCCATTGGGTAATAGCTCTGAG-3′; β-actin forward, 5′-CATGTACGTTGCTATCCAG GC-3′ and reverse, 5′-CTCCTTAATGTCACGCACGAT-3′; Cdkn1a forward, 5′-TGTCCGTCAGAACCCATGC-3′ and reverse, 5′-AAAGTCGAAGTTCCATCGCTC-3′. The mRNA expression of the target gene was calculated by the 2^-ΔΔCt^ method [9] or relative to β-actin expression. The PerfectStrat Green qPCR SuperMix was used to detect relative miRNA expression with 45 cycles of PCR thermocycling. The miRNA relative expression was calculated by the 2^-ΔΔCt^ method using U6 small nucleolar RNA gene as internal reference. The forward primer of miR-17-5p is 5′-GCTTCGCAAAGTGCTTACAGTGC-3′, and the forward primer of U6 is GCTTCGGCAGCACATATACTAAAAT. The reverse primer of qPCR for miRNA is provided in the TransScript miRNA First-Strand cDNA Synthesis SuperMix named as *Universal miRNA qPCR Primer.*

### Chromatin immunoprecipitation (ChIP) assays

ChIP assays were performed using a ChIP assay kit (Abcam, cat. ab117138) according to the manufacturer’s instructions. Briefly, cells were fixed for 8 min with 1% free formaldehyde (Cell Signaling Technology, cat. no. 12606) and then disrupted in SDS lysis buffer. Chromatin was sonicated by an M220 Focused-ultrasonicator (Covaris, Inc.) to shear DNA to an average length ranging from 200 to 1000 bp, as verified by agarose gel electrophoresis. Next, chromatin was immunoprecipitated with antibodies (2 μL) directed against Stat5a, and 0.8 μL of nonimmune IgG was used as the negative control. Final DNA extractions were quantitative-PCR amplified using primer pairs that cover the sequence in the Dnmt3a promoter region (− 2860 bp to + 140 bp). Sequences of the primers for quantitative PCR are listed in Table 2. For the primers numbered Dnmt3a-1F and Dnmt3a-1R, the amplified fragments are numbered ChIP1, and so on.

**Table 2:** Primers for quantitative PCR of the Dnmt3a promoter region

Number	Primer	Sequences (5′-3′)
1	Dnmt3a-1F	GGAATGAGGTGGAGTCCTGA
	Dnmt3a-1R	CATGGCCATAGGACAGAGGT
2	Dnmt3a-2F	ACCTCTGTCCTATGGCCATG
	Dnmt3a-2R	CCCCAGTCCCTGACAGTG
3	Dnmt3a-3F	CACTGTCAGGGACTGGGG
	Dnmt3a-3R	GTGGGTCACGACCTCTTTGA
4	Dnmt3a-4F	TCAAAGAGGTCGTGACCCAC
	Dnmt3a-4R	GGGCACTTTCTCCCTGAAGT
5	Dnmt3a-5F	ACTTCAGGGAGAAAGTGCCC
	Dnmt3a-5R	TGTCAGGGGACTGAGCTCTT
6	Dnmt3a-6F	AAGAGCTCAGTCCCCTGACA
	Dnmt3a-6R	CACTGTTAGAGCAATCGGATGA
7	Dnmt3a-7F	TAGCCCTGGCTGTATTGGA
	Dnmt3a-7R	GCCTGAATTTGATTCCCAGA
8	Dnmt3a-8F	TTTTGTGCCTGGTCTCCTTC
	Dnmt3a-8R	AGCTTGATGGCAGAGTGCTT

### Statistical analysis

The statistical analyses were performed with GraphPad Prism 7 software. The results were from triplicate experiments, and the data are presented as the mean ± SD (standard deviation). The significance of mean values was determined by unpaired two-tailed Student’s t test (*p*-value cut-offs: ****p<0.0001, ***p<0.001, **p<0.01, *p<0.05) and the Type I error for all statistical tests is α = 0.05.

## Results

### Dnmt3a expression is repressed by Stat5a at the transcriptional and translational levels in Jak2^V617F^ positive BaF3 cells and bone marrow mononuclear cells of cMPN patients

**Fig. 1:**
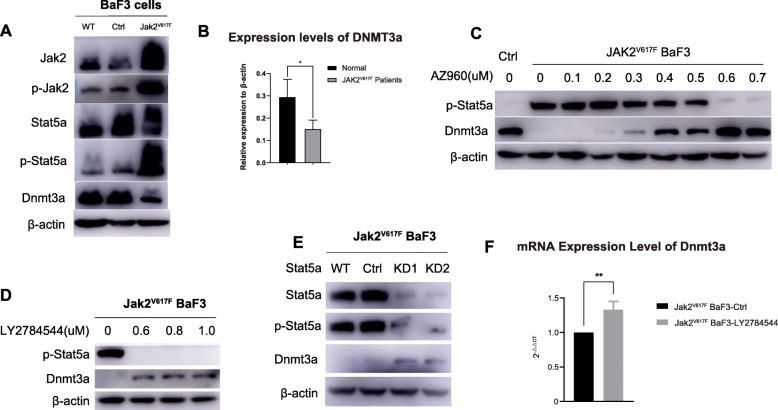
Aberrant activation of the JAK-STAT pathway represses Dnmt3a transcription and translation via Stat5a. A. The expression level of Jak2, p-Jak2, Stat5a, p-Stat5a and Dnmt3a in wild-type and Jak2^V617F^ BaF3 cells. Full-length blot images are presented in Supplementary Fig. 1. B. Transcription level of DNMT3a in bone marrow mononuclear cells from JAK2^V617F^ MPN patients and normal controls. C. p-Stat5a and Dnmt3a of Jak2^V617F^ Baf3 cells incubated with AZ960. Full-length blot images are presented in Supplementary Fig. 2. D. p-Stat5a and Dnmt3a of Jak2^V617F^ Baf3 cells incubated with LY2784544. Full-length blot images are presented in Supplementary Fig. 3. E. In Jak2^V617F^ BaF3 cells, the protein expression level of Dnmt3a after Stat5a was knocked down. Full-length blot images are presented in Supplementary Fig. 4. F. The transcriptional level of Dnmt3a in Jak2^V617F^ Baf3 cells with or without LY2784544 incubation

In this study, we mimicked JAK2^V617F^ mutation-positive cMPNs in vitro by overexpressing the murine Jak2 gene with the V617F mutation in the mouse pro-B cell line BaF3 and used wild-type BaF3 cells transfected with empty vector as controls. Firstly, in Jak2^V617F^ BaF3 cells, the JAK-STAT pathway was abnormally activated, which resulted in the elevated expression of the Jak2, p-Jak2, Stat5a and p-Stat5a proteins (Fig. 1A; Supplementary Fig. 1). Moreover, we found that the expression level of Dnmt3a protein was decreased in Jak2^V617F^ BaF3 cells compared with control BaF3 cells (Fig. 1A; Supplementary Fig. 1). Bone marrow mononuclear cells were isolated from JAK2^V617F^ cMPN patients and the normal controls. The expression of DNMT3a in bone marrow mononuclear cells were detected by RT-qPCR, and the results showed that DNMT3a was less transcribed in cMPN patients with JAK2^V617F^ mutation compared with the normal controls (Fig. 1B), which was consistent with the result observed in Jak2^V617F^ BaF3 cells. We then incubated Jak2^V617F^ BaF3 cells with two JAK-STAT pathway inhibitors, AZ960 and LY2784544, to verify that the decreased protein level of Dnmt3a was indeed associated with abnormal activation of the Jak-Stat pathway. It showed that the expression level of Dnmt3a increased gradually with the gradual suppression of p-Stat5a expression, indicating that the protein levels of Dnmt3a was truely negatively regulated by activated Jak-Stat pathway especially activated Stat5a (Fig. 1C and Supplementary Fig. 2; Fig. 1D and Supplementary Fig. 3). To avoid the off-target effect of Jak-Stat pathway inhibitors, Stat5a-knockdown stable Jak2^V617F^ BaF3 cell lines was also constructed and the elevated protein expression level of Dnmt3a was observed in this stable cell line (Fig. 1E and Supplementary Fig. 4). RT-qPCR assays showed that inhibition of Stat5a by LY2784544 incubation led to upregulation of Dnmt3a transcriptional levels (Fig. 1F). These results firmed from different aspects that the Dnmt3a was negatively regulated in Jak2^V617F^ positive cells owing to the abnormal activation of Jak-Stat pathway caused by Jak2^V617F^ mutation.

### Experiments in myeloid tumor cell lines further confirm that the Dnmt3a expression is repressed by Stat5a and Stat5a is the upstream regulator of Dnmt3a

**Fig. 2:**
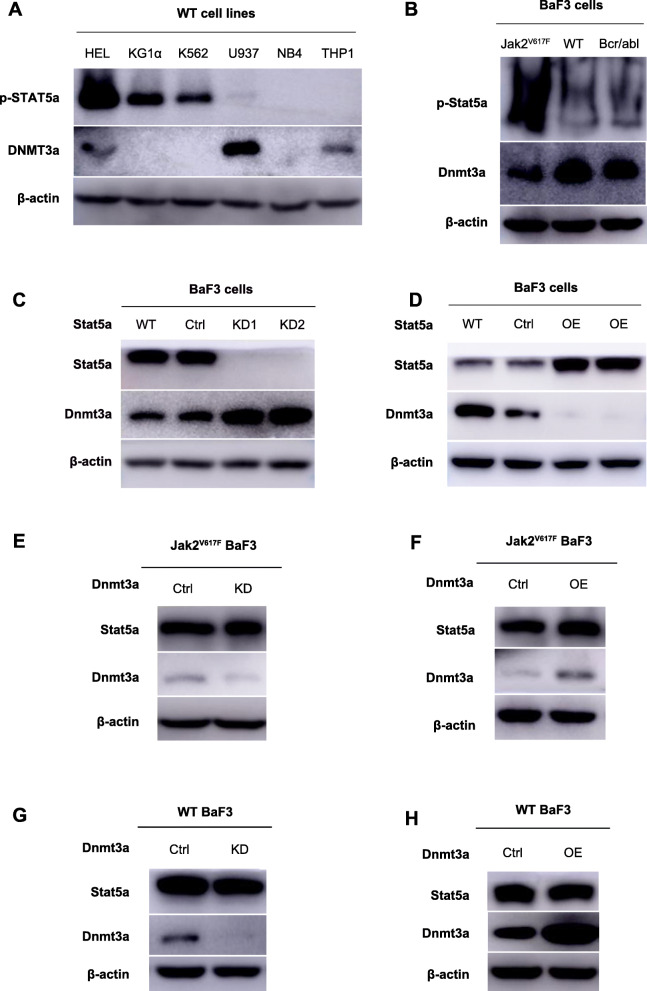
Dnmt3a expression is repressed by Stat5a and Stat5a is the upstream regulator of Dnmt3a. A. The expression levels of p-STAT5a and DNMT3a proteins in human myeloid tumor cell lines. Full-length blot images are presented in Supplementary Fig. 5. B. The expression levels of p-Stat5a and Dnmt3a proteins in murine myeloid tumor cell lines. Full-length blot images are presented in Supplementary Fig. 6. C. In wild type BaF3 cells, the protein expression level of Dnmt3a after Stat5a was knocked down. Full-length blot images are presented in Supplementary Fig. 7. D. In wild type BaF3 cells, the protein expression level of Dnmt3a after overexpression of Stat5a. Full-length blot images are presented in Supplementary Fig. 8. E & F. In Jak2^V617F^ BaF3 cells, the protein expression of Stat5a after knockdown or overexpression of Dnmt3a. Full-length blot images are presented in Supplementary Fig. 9 and Supplementary Fig. 10. G & H. In wild-type BaF3 cells, the protein expression of Stat5a after knockdown or overexpression of Dnmt3a. Full-length blot images are presented in Supplementary Fig. 11 and Supplementary Fig. 12

In order to supply more evidence to prove that the Dnmt3a is downregulated by Stat5a, the expression levels of p-STAT5a and DNMT3a proteins in other human or murine myeloid tumor cell lines were examined, as shown in Fig. 2A (Supplementary Fig. 5) and Fig. 2B (Supplementary Fig. 6). In Fig. 2A, we selected six human myeloid tumor cell lines, of which the HEL cell line is a human erythroleukemia cell line with the JAK2^V617F^ mutation, while all other cell lines do not carry the JAK2^V617F^ mutation. The p-STAT5a expression level was most significant in the HEL cell line, indicating that the JAK-STAT pathway was activated in HEL cells. In HEL, KG1α and K562 cells, the expression levesl of p-STAT5a were higher and the expression levels of DNMT3a protein were correspondingly lower. In contrast, p-STAT5a expression levels were lower in U937 and THP1 cells, and the protein expression levels of DNMT3a were higher. In Fig. 2B, we selected wild-type BaF3 cells, BaF3 cells with the Bcr-abl fusion gene and BaF3 cells with the Jak2^V617F^ mutation to detect the expression levels of p-Stat5a and Dnmt3a. Similar to the aforementioned assay, p-Stat5a expression was the highest in Jak2^V617F^ BaF3 cells, while the expression level of Dnmt3a was correspondingly the lowest.

To further confirm that this effect is mediated by the Stat5a protein, Stat5a knockdown and overexpression cell lines using wild-type BaF3 cells were constructed as shown in Fig. 2C (Supplementary Fig. 7) and Fig. 2D (Supplementary Fig. 8). In wild-type BaF3 cells without Jak2^V617F^ mutation, the protein expression level of Dnmt3a increased as Stat5a was knocked down. Conversely, the protein expression level of Dnmt3a decreased with overexpression of Stat5a. These results indicated that the Stat5a protein is the key factor affecting the expression of Dnmt3a protein, and the inhibition of Dnmt3a in Jak2^V617F^ BaF3 cells is caused by abnormal activation of Stat5a.

We also altered the expression level of Dnmt3a in Jak2^V617F^ BaF3 cells (Fig. 2E and Supplementary Fig. 9; Fig. 2F and Supplementary Fig. 10) and wild-type BaF3 cells (Fig. 2G and Supplementary Fig. 11; Fig. 2H and Supplementary Fig. 12), and found that neither Dnmt3a knockdown nor overexpression had any effect on the protein expression of Stat5a, which further verified that Stat5a was the upstream regulator of Dnmt3a.

In conclusion, all of the above results confirmed that the Dnmt3a is negatively regulated by the Stat5a protein and Stat5a is the upstream regulator of Dnmt3a.

### The Stat5a protein binds with the GAS motif region of Dnmt3a promoter and suppresses the transcriptional activity of Dnmt3a promoter

**Fig. 3:**
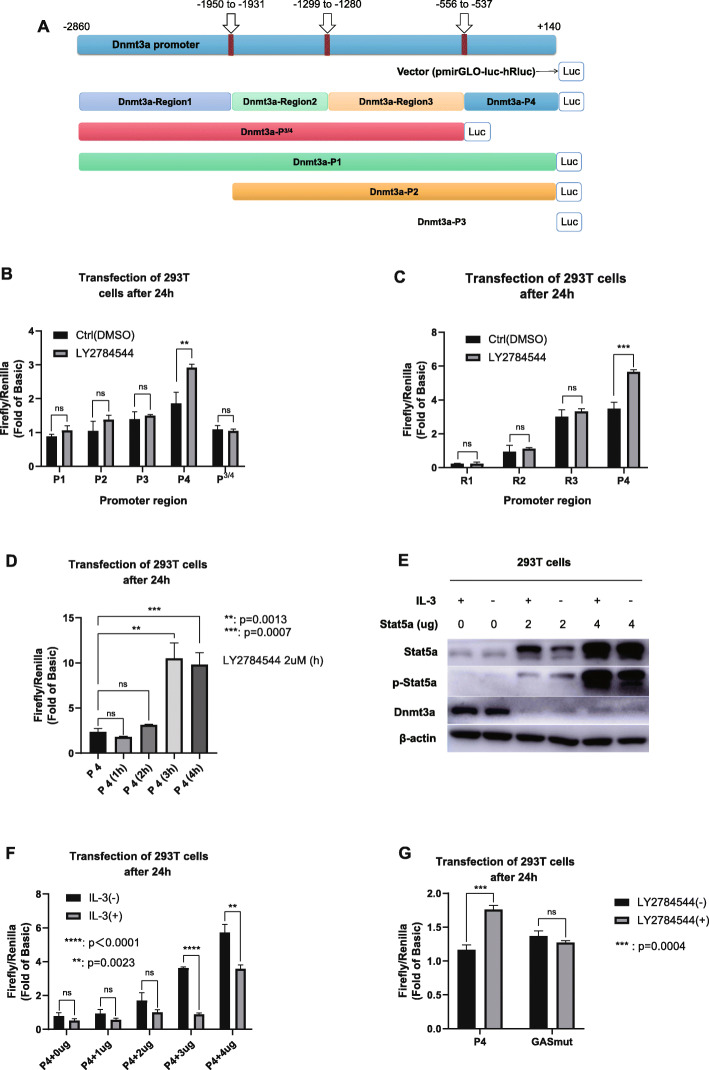
Aberrant activation of Stat5a downregulates the promoter transcriptional activity of Dnmt3a. A. Segments of the promoter region of the Dnmt3a gene. B & C. Incubation with LY2784544 significantly increased the fluorescence intensity of 293 T cells transfected with the P4 region luciferase plasmid. D. The P4 region promoter transcriptional activity was strongest after 3 h of LY2784544 incubation. E. Stat5a was overexpressed in 293 T cells, and IL-3 was added to simulate the abnormal activation and phosphorylation of Stat5a. Full-length blot images are presented in Supplementary Fig. 13. F. Overexpression and activation of Stat5a inhibited the transcriptional activity of the P4 region promoter. G. The effect of LY2784544 on transcriptional activity of the Dnmt3a promoter with or without mutated GAS

To investigate the mechanism by which Stat5a regulates the transcriptional level of Dnmt3a, we selected the region approximately 3000 bp upstream of the first exon of the Dnmt3a gene for amplification and constructed dual-luciferase plasmids with different truncation ranges, named them respectively as P1, P2, P3, P4, P^3/4^, Region1, Region2, and Region3 (Fig. 3A). After incubation with the Stat5a inhibitor LY2784544 for 2 h, it was found that the fluorescence intensity was significantly increased in 293 T cells transfected with the P4 region luciferase plasmid, while in the 293 T cells transfected with the P^3/4^ plasmid which except for the P4 region, showed no significant change in fluorescence intensity after incubation with LY2784544 for the same time (Fig. 3B). Furthermore, LY2784544 incubation of 293 T cells transfected with the P1, P2, P3, R1, R2 and R3 regions of Dnmt3a promoter showed no significant change in relative fluorescence intensity (Fig. 3B and Fig. 3C). These results indicated that inhibition of the protein level of Stat5a significantly increased the transcriptional activity of the Dnmt3a promoter, and we hypothesized that promoter region of Dnmt3a might locate at P4, which was located in the region approximately 800 bp upstream of the first exon. Next, we examined the time effect of inhibitor incubation on the fluorescence intensity. The results showed that the P4 region promoter transcriptional activity was the strongest after 3 h of inhibitor incubation (Fig. 3D). Thereafter, by overexpressing Stat5a in 293 T cells and adding IL-3 to simulate the abnormal activation and phosphorylation of Stat5a, we found that overexpression and activation of Stat5a inhibited the transcriptional activity of the P4 region promoter (Fig. 3E and Supplementary Fig. 13; Fig. 3F), indicating that the activation and phosphorylation of Stat5a did inhibit the transcriptional activity of Dnmt3a promoter, which will in turn induce downregulation of the protein expression level of Dnmt3a.

To investigate the specific site of the Dnmt3a promoter at which Stat5a binds to, we predicted it using the JASPAR database (http://jaspar.genereg.net/). The gamma-activated sequence motif (GAS) which was well reported to be binded with STAT5a protein [10] is found to be located at the P4 promoter region of Dnmt3a. So we next mutated the GAS motif sequence in Dnmt3a from TTCTGGGAA to TAAACCTGG to construct a dual-luciferase plasmid of the mutated P4 region (named GASmut). As shown in Fig. 3G, the transcriptional activity of P4 promoter region was significantly higher when cells were incubated with LY2784544. However, after the GAS motif in P4 was mutated, the LY2784544 incubation failed to elevate the transcriptional activity of P4 promoter region. The above results suggest that Stat5a negatively regulates Dnmt3a probably by regulating the transcriptional activity of the promoter, and the specific binding site of regulation may be within the GAS motif and its nearby regions.

**Fig. 4:**
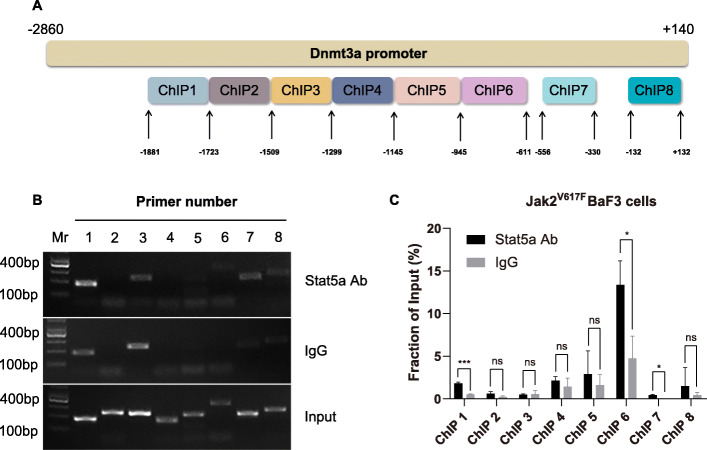
Stat5a downregulates Dnmt3a protein expression by directly binding to the Dnmt3a promoter. A. The schema of the 8 regions of ChIP analysis; B & C. Stat5a protein occupied several regions (ChIP1, ChIP6 and ChIP7) of the Dnmt3a promoter. Full-length blot images are presented in Supplementary Fig. 14

Then, we performed ChIP assays to find out whether Stat5a protein binds to the promoter of Dnmt3a directly. qPCR primers were designed to cover the full-length sequence of 3000 bp upstream of the first exon of Dnmt3a, with a schematic diagram shown in Fig. 4A. Results of ChIP assays (Fig. 4B and C, Supplementary Fig. 14) identified that the Stat5a protein occupied several regions of the Dnmt3a promoter (ChIP1, ChIP6 and ChIP7). In addition, the GAS site in Fig. 3 was located in the ChIP7 fragment (− 556 bp ~ − 330 bp), which further confirmed our hypothesis that the promoter region of Dnmt3a might locate in approximately 800 bp upstream of the first exon, and indicated that Stat5a regulated the transcriptional activity of the Dnmt3a promoter by occupying the GAS motif and its nearby regions of the promoter.

Taken together, these results demonstrated that the direct binding between Stat5a protein and Dnmt3a promoter mediated the inhibited transcription and expression of Dnmt3a.

### Dnmt3a suppresses the proliferation capacity of Jak2^V617F^ BaF3 cells

**Fig. 5:**
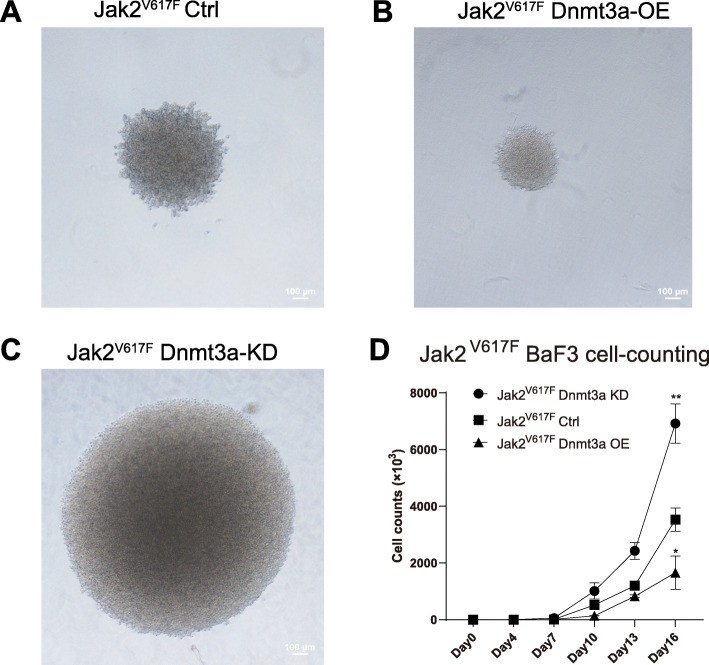
Knockdown or overexpression of Dnmt3a in Jak2^V617F^ BaF3 cells significantly affects cell proliferation capacity. A. Soft agar clone formation of Jak2^V617F^ BaF3 cells; B. Soft agar clone formation of Dnmt3a-overexpressing Jak2^V617F^ BaF3 cells; C. Soft agar clone formation of Dnmt3a-knockdown Jak2^V617F^ BaF3 cells; D. Cell proliferation level of Jak2^V617F^ BaF3 cells with Dnmt3a overexpression or with Dnmt3a knockdown

To investigate the effect of Dnmt3a on the proliferation ability of Jak2^V617F^ BaF3 cells, we constructed Dnmt3a silencing and overexpression plasmids, and the corresponding stably transformed Jak2^V617F^ BaF3 cell lines were constructed using lentiviral infection techniques. The in vitro clonogenic ability was detected by soft agar clone formation, and we found that the clonogenic ability of Jak2^V617F^ BaF3 cells with Dnmt3a overexpression was diminished (Fig. 5A and Fig. 5B), while the clonogenic ability of Jak2^V617F^ BaF3 cells with Dnmt3a knockdown was enhanced (Fig. 5A and Fig. 5C). Then, we performed cell counting assays on stably transduced Jak2^V617F^ BaF3 cells with Dnmt3a knockdown or overexpression. As shown in Fig. 5D, the proliferation level of Jak2^V617F^ BaF3 cells overexpressing Dnmt3a was reduced, while the proliferation ability of Jak2^V617F^ BaF3 cells with Dnmt3a knockdown was enhanced. In conclusion, the above results indicate that Dnmt3a significantly downregulates the proliferation capacity of Jak2^V617F^ cells.

### Dnmt3a mediates G0/G1 arrest by positive-regulating the Cdkn1a expression in Jak2^V617F^ cells

**Fig. 6:**
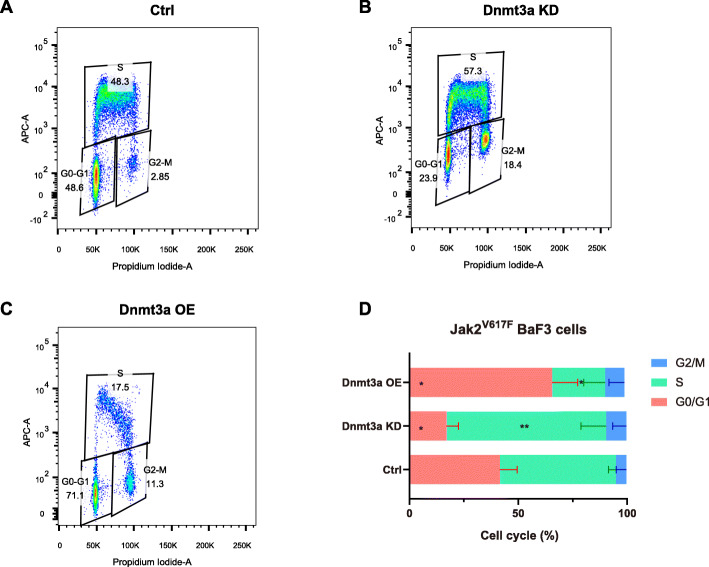
Dnmt3a regulates the cell cycle of Jak2^V617F^ BaF3 cells by regulating Cdkn1a expression. A. Cell cycle distribution of Jak2^V617F^ BaF3 cells. B. Cell cycle distribution of Dnmt3a-knockdown Jak2^V617F^ BaF3 cells. C. Cell cycle distribution of Dnmt3a-overexpressing Jak2^V617F^ BaF3 cells. D. Cell cycle distribution statistics of each group (A-C) of Jak2^V617F^ BaF3 cells

To clarify how Dnmt3a suppresses the proliferation capacity of Jak2^V617F^ cells, we performed cell cycle distribution analysis. As shown in Fig. 6A-Fig. 6D, compared with the control Jak2^V617F^ BaF3 cells, Dnmt3a-knockdown (Dnmt3a KD) Jak2^V617F^ BaF3 cells had significantly increased S-phase cell proportion and decreased G0/G1-phase cell proportion, while Dnmt3a overexpression (Dnmt3a OE) Jak2^V617F^ BaF3 cells had significantly increased G0/G1-phase cell proportion and decreased S-phase cell proportion. These results indicates that Dnmt3a can inhibit the cell cycle progression of Jak2^V617F^ BaF3 cells, mediates G0/G1 arrest and results in the suppression of the proliferation of Jak2^V617F^ BaF3 cells at last.

**Fig. 7:**
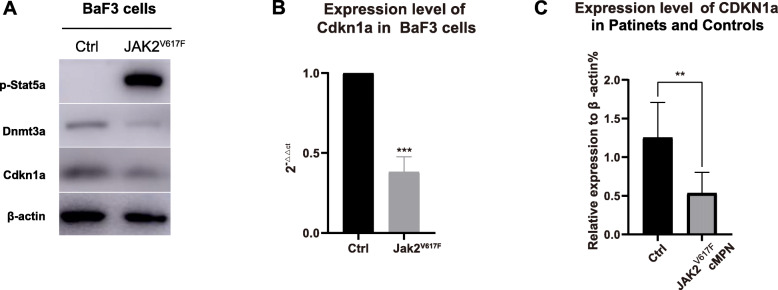
The expression level of Cdkn1a is downregulated in Jak2^V617F^ cells. A. The expression level of Cdkn1a was significantly suppressed in Jak2^V617F^ BaF3 cells compared with control BaF3 cells. Full-length blot images are presented in Supplementary Fig. 15. B. The transcription level of Cdkn1a was significantly suppressed in Jak2^V617F^ BaF3 cells compared with control BaF3 cells. C. The transcription level of Cdkn1a is lower in JAK2^V617F^ cMPN patients than that in normal controls

**Fig. 8:**
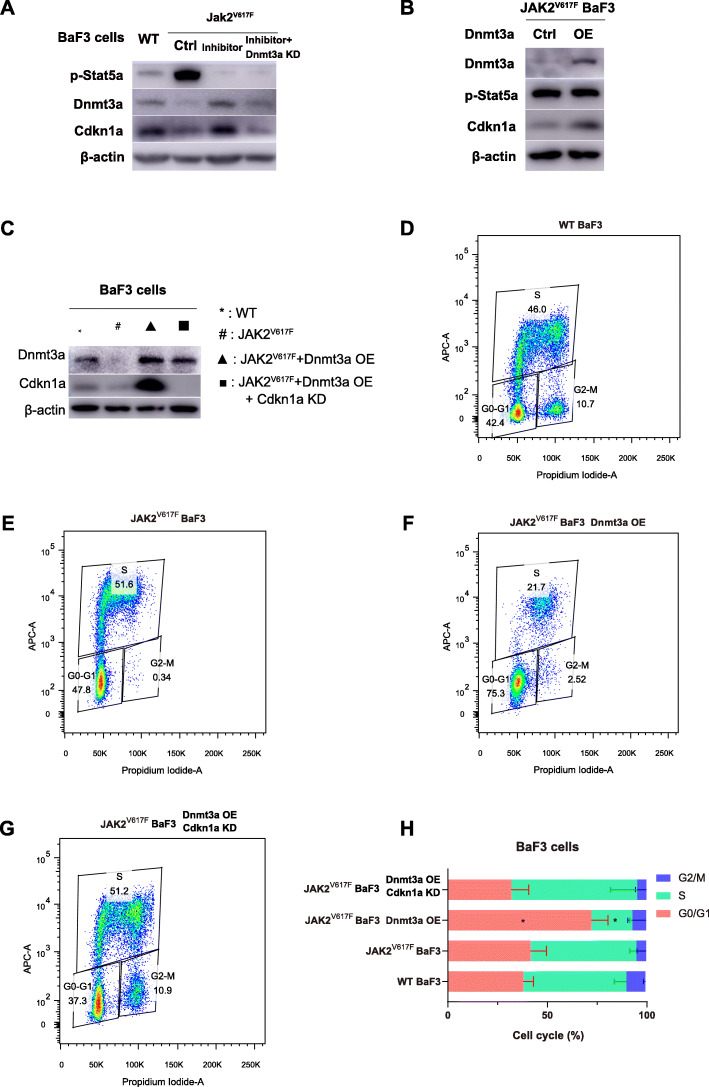
Dnmt3a regulates the cell cycle of Jak2^V617F^ Baf3 cells through Cdkn1a. A & B. Dnmt3a induced Cdkn1a expression in Jak2^V617F^ BaF3 cells. Full-length blot images are presented in Supplementary Fig. 16 and Supplementary Fig. 17. Inhibitor: LY2784544 incubation. C. Jak2^V617F^ Baf3 cells with Dnmt3a overexpression and with both Dnmt3a overexpression and Cdkn1a knockdown were established. Full-length blot images are presented in Supplementary Fig. 18. D. Cell cycle distribution of wild-type BaF3 cells. E. Cell cycle distribution of Jak2^V617F^ BaF3 cells. F. Cell cycle distribution of Jak2^V617F^ BaF3 cells with Dnmt3a overexpression. G. Cell cycle distribution of Jak2^V617F^ BaF3 cells with both Dnmt3a overexpression and Cdkn1a knockdown. H. Cell cycle distribution statistics of each group of Jak2^V617F^ BaF3 cells

How does Dnmt3a mediate G0/G1 arrest? In order to answer this question, we firstly tested the expression of cell cycle-related gene Cdkn1a. The Cdkn1a gene encodes a potent cyclin-dependent kinase inhibitor that binds to and inhibits the activity of cyclin-dependent kinase 2 or the cyclin-dependent kinase 4 complex, therefore plays a role in regulating the progression of the cell cycle in the G1 phase, causing the G1 phase of the cell cycle to arrest [11]. We found that the expression level of Cdkn1a was significantly suppressed in Jak2^V617F^ BaF3 cells compared with control BaF3 cells (Fig. 7A and Supplementary Fig. 15; Fig. 7B). Furthermore, the expression of Cdkn1a in JAK2^V617F^ cMPN patients and normal controls were examined and the results showed that Cdkn1a expression levels in JAK2^V617F^ cMPN patients were lower than that in normal controls, which was consistent with the results in BaF3 cell models (Fig. 7C). Collectively, the above results show that the expression of the Cdkn1a is downregulated in Jak2^V617F^ cells. Secondly, we explored the role of Dnmt3a in the downregulation of Cdkn1a. We detected the expression level of Cdkn1a in several different groups of Jak2^V617F^ Baf3 cells, as shown in Fig. 8A (Supplementary Fig. 16). When incubated with LY2784544, the inhibitor of Stat5a, the expression of Dnmt3a was elevated, and the expression of Cdkn1a increased simultaneously. Furthermore, when Dnmt3a was knocked down, the expression of Cdkn1a was correspondingly reduced. Besides, the expression level of Cdkn1a was observed to increase in Jak2^V617F^ BaF3 cells overexpressing Dnmt3a (Fig. 8B and Supplementary Fig. 17). Thus, we concluded that Dnmt3a can positively regulates the expression of the Cdkn1a gene in Jak2^V617F^ BaF3 cells.

We also established three types of stable cell lines to further study the relationship among Jak2^V617F^, Dnmt3a and Cdkn1a: the Jak2^V617F^ BaF3 cell line (Jak2^V617F^), the Jak2^V617F^ BaF3 with Dnmt3a overexpression (Jak2^V617F^ + Dnmt3a OE) cell line, and the Jak2^V617F^ BaF3 with both Dnmt3a OE and Cdkn1a knockdown (Jak2^V617F^ + Dnmt3a OE + Cdkn1a KD) cell line (Fig. 8C and Supplementary Fig. 18). The cell cycle analysis was performed and the results were shown in Fig. 8D-Fig. 8G. The cell cycle distribution of wild-type BaF3 cells (WT) and Jak2^V617F^ BaF3 cells (Jak2^V617F^) showed no significant differences in cell cycle (Fig. 8D and Fig. 8E). The Dnmt3a-overexpressing Jak2^V617F^ BaF3 cells showed obvious cell cycle blockade in the G0/G1 phase (Fig. 8F) and after we knocked down Cdkn1a expression in Dnmt3a-overexpressing Jak2^V617F^ BaF3 cells, the G0/G1 cell cycle blockade was eliminated and showed no significant difference compared with Jak2^V617F^ BaF3 cells (Fig. 8G and H). The results indicated that Dnmt3a can cause the cell cycle blockade in Jak2^V617F^ cells and Dnmt3a carries out this function via Cdkn1a-related pathway.

In summary, all of the experiments above elucidated from different aspects that Dnmt3a suppresses the proliferation capacity of Jak2^V617F^ cells by mediating G0/G1 arrest via positive-regulating the Cdkn1a expression in Jak2^V617F^ cells.

### Dnmt3a negatively regulates miR-17-5p and miR-17-5p represses Cdkn1a expression

**Fig. 9:**
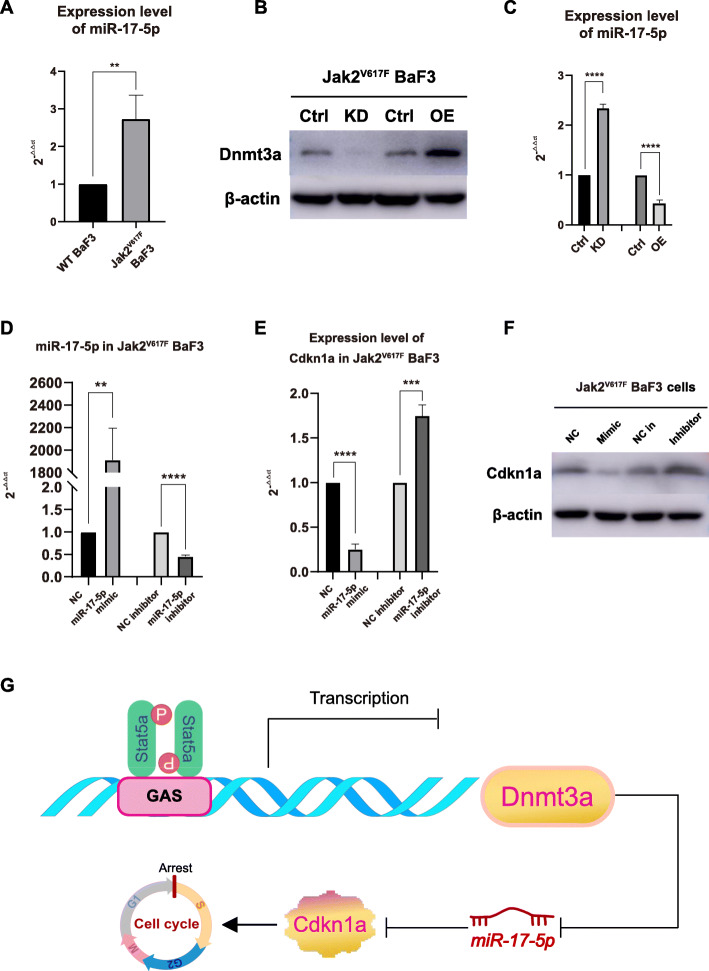
Dnmt3a negatively regulates miR-17-5p and miR-17-5p represses Cdkn1a expression in Jak2^V617F^ BaF3 cells. A. The expression level of miR-17-5p in Jak2^V617F^ BaF3 cells and control BaF3 cells. B. The verification of the knockdown and overexpression effect of Dnmt3a in Jak2^V617F^ BaF3 cells. Full-length blot images are presented in Supplementary Fig.19. C. The expression level of miR-17-5p in Jak2^V617F^ BaF3 cells with Dnmt3a knockdown and overexpression. D. The verification of the effect of transfection of miR-17-5p mimic and inhibitor in Jak2^V617F^ BaF3 cells. E & F. The transcription and expression of Cdkn1a in Jak2^V617F^ BaF3 cells transfected with miR-17-5p mimic or inhibitor. Full-length blot images are presented in Supplementary Fig.19. G. Graphic abstract of the present study

It has been reported that Dnmt3a suppresses the miR-17-5p in mouse models [12] and there is a conserved site for miR-17-5p at the 3’UTR of mouse Cdkn1a with high context score (98) and 7 mer seed match (Table 3) according to TargetScanMouse database (http://www.targetscan.org /mmu_72/). Thus we suspect that miRNA may play a role in the regulation of Cdkn1a by Dnmt3a. To confirm the hypothesis, we detected the expression level of miR-17-5p in Jak2^V617F^ BaF3 cells, control BaF3 cells, Jak2^V617F^ BaF3 cells with Dnmt3a overexpression and Jak2^617F^ BaF3 cells with Dnmt3a knockdown. It was observed that the expression of miR-17-5p was higher in Jak2^V617F^ BaF3 cells than in control BaF3 cells (Fig. 9A). The expression level of miR-17-5p in Jak2^V617F^ BaF3 cells was also higher than the Jak2^V617F^ BaF3 cells with Dnmt3a overexpression, but lower than that in Jak2^V617F^ BaF3 cells with Dnmt3a knockdown (Fig. 9B and C, Supplementary Fig. 19). These observation indicated that expression of miR-17-5p is negatively correlated with Dnmt3a, and Dnmt3a might negatively regulates the expression of miR-17-5p. The decreased expression of Dnmt3a was accompanied by the increased expression of miR-17-5p due to the reduced inhibition of Dnmt3a on miR-17-5p in Jak2^V617F^ BaF3 cells.

**Table 3:** miR-17-5p target sequences of Cdkn1a mRNA 3′-UTR

	Sequence
miR-17-5p	3′-GAUGGACGUGACAUU**CGUGAAA**C-5’
Cdkn1a 3’UTR	5′-CCUCAGACCUGAAUA**GCACUUU**G-3’

Then miR-17-5p mimic and inhibitor were transfected into Jak2^V617F^ BaF3 cells and the expression alteration were confirmed by RT-qPCR. In Jak2^V617F^ BaF3 cells transfected with miR-17-5p mimic, the transcriptional and translational levels of Cdkn1a was decreased (Fig. 9D-F, Supplementary Fig. 19). Consistently, in Jak2^V617F^ BaF3 cells transfected with miR-17-5p inhibitor, the transcriptional and translational levels of Cdkn1a was increased (Fig. 9D-F, Supplementary Fig. 19). These results indicated that Cdkn1a is a downstream target of miR-17-5p and inhibited by miR-17-5p in Jak2^V617F^ BaF3 cells.

In summary, as miR-17-5p inhibits the expression of Cdkn1a and Dnmt3a downregulats the expression of miR-17-5p in Jak2^V617F^ BaF3 cells, the mechanism under which Cdkn1a expression is changed in Dnmt3a expression-altered cells might be Dnmt3a attenuate the inhibition of Cdkn1a mediated by miR-17-5p. Finally, the graphic abstract of the present study was shown in Fig. 9G.

## Discussion

DNA methylation is an important epigenetic mechanism that interferes tumogenesis [13]. Mutations in the gene encoding DNMT3a were reported in patients with various hematological malignancies, pointing to DNMT3a as a critically important new tumor suppressor [14–16]. Our study found that abnormally activated Stat5a inhibits the transcription and translation of Dnmt3a by occupying the GAS motif of the promoter of Dnmt3a and downregulating the transcriptional activity of the promoter of Dnmt3a. Dnmt3a in Jak2^V617F^ cells positively regulates the expression of Cdkn1a via miR-17-5p and significantly affects cell proliferation, mediating G0/G1 cell cycle arrest in Jak2^V617F^ BaF3 cells. Therefore, we speculate that the axis of Stat5a/Dnmt3a/miR-17-5p/Cdkn1a identified in this study in JAK2^V617F^-positive cells might play an important role in cMPNs and potentially provides a treatment target for cMPNs.

The somatic JAK2^V617F^ mutation is well known to play a crucial role in classical myeloproliferative neoplasms. JAK2^V617F^ mutation was reported to promote the pathogenesis and progression of hematological tumors by regulating downstream target genes. For example, expression of HIF-1α was elevated in Jak2^V617F^-expressing 32D cells, and inhibition of the binding of HIF-1 to hypoxia response elements (HREs) with echinomycin impairs the growth and survival of Jak2^V617F^-positive 32D cells [17]. The JAK2^V617F^ mutation also enforced Mcl-1 transcription to sensitize myelodysplastic syndrome cell lines to apoptosis induced by a Bcl-xL/Bcl-2 inhibitor [18]. While the mutation of DNMT3a is known to play an important role in the pathogenesis of cMPN and a synergistic carcinogenic effect is known to exist for cMPNs driven by JAK2^V617F^ and DNMT3a deletion, how the Dnmt3a is regulated by Jak2^V617F^ mutation still needs research. Here, we found that in Jak2^V617F^ BaF3 cells, the expression of Dnmt3a was inhibited compared with the wild-type BaF3. Further investigation of Stat5a inhibitor and Stat5a knockdown or overexpression found that the reduction in Dnmt3a in Jak2^V617F^ BaF3 cells was mediated by abnormal activation of Stat5a. Subsequently, we overexpressed and knocked down Stat5a in wild-type BaF3 cells and found that as the expression of the Stat5a protein increased or decreased, the expression of Dnmt3a decreased or increased, respectively. Therefore, our research shows that the inhibition of Dnmt3a expression is mediated by abnormally activated Stat5a protein, indicating that Dnmt3a is a target gene of Stat5a in Jak2^V617F^ BaF3 cells.

Signal transducer and activator of transcription (STAT) proteins reportedly bind to palindromic sites to regulate target gene expression [19, 20], and STAT5 transcription factors take part in the formation of DNA-protein complexes [21]. In the present study, dual-luciferase reporter vector assays were performed to identify the promoter region of Dnmt3a. Incubation with a Stat5a inhibitor significantly induced the promoter activity of the Dnmt3a promoter, while overexpression and activation of Stat5a reduced the promoter activity of the Dnmt3a promoter. Furthermore, after GAS in the Dnmt3a promoter region was mutated, incubation with the Stat5a inhibitor did not influence the activity of the mutated promoter. ChIP assays of Stat5a on the Dnmt3a promoter further demonstrated that Stat5a binds to the promoter region of Dnmt3a. Taken together, these results represent an important and critical discovery of our study; that is, Stat5a inhibits Dnmt3a expression by directly occupying promoter region of Dnmt3a. As shown in Fig. 4, Stat5a also occupied the ChIP6 region and there is a GAS site (TTCCAGGAA) in ChIP6 region, but according to the results of Fig. 3B and 3C, neither transcription activity of P3 nor Region3 was altered after incubation of LY2784544, and ChIP6 is located at Region3. It’s one of limitations of the study that the effect of mutation of GAS site at ChIP6 region was not examined and the reason why transcription activity of P3 nor Region3 was altered after incubation of LY2784544 was not explored. Our further study will follow up persistently.

DNMT3a belongs to a family of highly conserved DNA methyltransferases that catalyze 5-methylcytosine methylation and methylate DNA at unique sites as well as at repetitive elements [14]. It was reported that hematopoietic-specific Dnmt3a loss in mice leads to enhanced stem-cell self-renewal at the expense of differentiation and predisposes mice to the acquisition of cooperating proleukemogenic mutations in the expanded clone [22]. In our study, we found that knockdown of Dnmt3a in Jak2^V617F^ BaF3 cells significantly inhibited cell proliferation and induced G0/G1 phase cell cycle arrest. Our results showed that Dnmt3a might mediate G0/G1 phase cell cycle arrest by positively regulating the G1/S checkpoint marker Cdkn1a. It has been reported that compared with AML patients with FLT3 mutations alone, AML patients with mutations of both DNMT3A and FLT3 showed homeobox gene overexpression and enhancer hypomethylation, which resulted in a lower enrichment score of G2/M checkpoint genes [23]. Our study demonstrated that in JAK2^V617F^ cMPNs, Dnmt3a positively regulates the expression of Cdkn1a, which induces G0/G1 cell cycle arrest, indicating that Dnmt3a also serves as a tumor suppressor in cMPNs by regulating the cell cycle through Cdkn1a, which in turn affects the proliferative capacity of Jak2^V617F^ BaF3 cells. Although Dnmt3a is a de novo methyltransferase [24] and usually induces the downregulation of target genes [25], in some carcinomas, Dnmt3a and p53 are positively correlated and play an important role in disease progression [26, 27]. The p53-p21 axis arrested the cell cycle at G2/M and prompted partial EMT and fibrosis together with inflammation [28]. Our study found that Cdkn1a induced cell cycle arrest at G0/G1 in Jak2^V617F^ BaF3 cells, which was regulated by the expression of Dnmt3a. Futhermore, we found that miR-17-5p inhibits the expression of Cdkn1a and Dnmt3a downregulats miR-17-5p in Jak2^V617F^ BaF3 cells, thus the mechanism under which Cdkn1a expression is changed in Dnmt3a expression-altered cells might be Dnmt3a attenuate the inhibition of Cdkn1a mediated by miR-17-5p. However, the direct regulation mechanism between Dnmt3a and miR-17-5p has not been explored, which is one of limitation of the study, will be followed up persistently in our future study. The deficiency of lack of in vivo validation will also be addressed in our future studies.

## Conclusions

In conclusion, our research reveals that Dnmt3a is a downstream target of Stat5a, it is negatively regulated by the Stat5a through occupance of the promoter region of Dnmt3a, and that Dnmt3a induces cell cycle arrest at the G0/G1 phase in Jak2^V617F^ Baf3 cells through Cdkn1a, which is down-regulated by the miR-17-5p. Our discovery of the Stat5a/Dnmt3a/miR-17-5p/Cdkn1a axis existing in JAK2^V617F^ cells might shed new light on the pathogenesis of JAK2^V617F^-positive cMPNs and will inform new approaches to treatment.

## Supplementary Information

**Additional file 1: Table S1.** Clinical characteristics of 12 samples (six JAK2V617F-positive cMPNs patients and six normal controls), from which bone marrow mononuclear cells were isolated

**Additional file 2: Table S2.** Primer sequences of different promoter regions of Dnmt3a (− 2860 bp to + 140 bp)

**Additional file 3: Fig. S1.** Uncropped images of Fig. 1A

**Additional file 4: Fig. S2.** Uncropped images of Fig. 1C

**Additional file 5: Fig. S3.** Uncropped images of Fig. 1D

**Additional file 6: Fig. S4.** Uncropped images of Fig. 1E

**Additional file 7: Fig. S5.** Uncropped images of Fig. 2A

**Additional file 8: Fig. S6.** Uncropped images of Fig. 2B

**Additional file 9: Fig. S7**. Uncropped images of Fig. 2C

**Additional file 10: **Fig. **S8.** Uncropped images of Fig. 2D

**Additional file 11: Fig. S9.** Uncropped images of Fig. 2E

**Additional file 12: Fig. S10.** Uncropped images of Fig. 2F

**Additional file 13: Fig. S11.** Uncropped images of Fig. 2G

**Additional file 14: Fig. S12.** Uncropped images of Fig. 2H

**Additional file 15: Fig. S13.** Uncropped images of Fig. 3E

**Additional file 16: Fig. S14.** Uncropped images of Fig. 4B

**Additional file 17: Fig. S15. **Uncropped images of Fig. 7A

**Additional file 18: Fig. S16.** Uncropped images of Fig. 8A

**Additional file 19: Fig. S17.** Uncropped images of Fig. 8B

**Additional file 20: Fig. S18.** Uncropped images of Fig. 8C

**Additional file 21: Fig. S19.** Uncropped images of Fig. 9B and F

## Data Availability

The data used and/or analysed during the current study are available from the corresponding author on reasonable request.

## Abbreviations

AML: Acute myeloid leukemia
Cdkn1a: Cyclin dependent kinase inhibitor 1a
CDS: Coding DNA Sequence
ChIP: Chromatin immunoprecipitation
cMPNs: Classical myeloproliferative neoplasms
DMEM: Dulbecco’s modified Eagle’s medium
DMSO: Dimethyl sulfoxide
Dnmt3a: DNA methyltransferase 3 alpha
ET: Essential thrombocythemia
FLT3: Fms Related Receptor Tyrosine Kinase 3
GAS: Gamma-activated sequence motif
GFP: Green fluorescent protein
HIF-1α: Hypoxia inducible factor 1 subunit alpha
HSPC: Hematopoietic stem/progenitor cells
JAK2: Janus Kinase 2
Mcl-1: MCL1 Apoptosis Regulator, BCL2 Family Member
PMF: Primary myelofibrosis
Puro: Puromycin
PV: Polycythemia vera
RPMI: Roswell Park Memorial Institute
shRNA: short hairpin RNA
Stat5a: Signal transducer and activator of transcription 5a
