# Supplementary material for: Dnmt3a is downregulated by Stat5a and mediates G0/G1 arrest by suppressing the miR-17-5p/Cdkn1a axis in Jak2V617F cells
Source: BMC Cancer. 2021 Nov 13;21:1213. doi: 10.1186/s12885-021-08915-0 (PMC8590245; doi:10.1186/s12885-021-08915-0)
Supplement: Supplementary file 1 — Additional file 1: Table S1. Clinical characteristics of 12 samples (six JAK2V617F-positive cMPNs patients and six normal controls), from which bone marrow mononuclear cells were isolated [file 12885_2021_8915_MOESM1_ESM.docx]

**Table 1**

Clinical characteristics of 12 samples (six JAK2^V617F^-positive cMPNs patients and six normal controls), from which bone marrow mononuclear cells were isolated

| No. | JA2V617F Mutation | MPL Mutation | CALR Mutation | Sex | Age (years) | Diagnosis | White blood cell （×10^9/L） | Platelet count（×10^9/L） | Hemoglobin（g/L） | Ferritin（μg/L） | Enlarged liver or spleen (mm×mm) | Karyotype |
| --- | --- | --- | --- | --- | --- | --- | --- | --- | --- | --- | --- | --- |
| 1 | Negative | Negative | Negative | Male | 76 | - | 5.8 | 140 | 124 | 357 | - | Normal |
| 2 | Negative | Negative | Negative | Male | 61 | - | 4.1 | 180 | 135 | 71.9 | - | Normal |
| 3 | Negative | Negative | Negative | Female | 58 | - | 3.4 | 130 | 121 | 140.5 | - | Normal |
| 4 | Negative | Negative | Negative | Male | 45 | - | 5.62 | 157 | 152 | 215 | - | Normal |
| 5 | Negative | Negative | Negative | Female | 53 | - | 2.87 | 276 | 112 | 508 | - | Normal |
| 6 | Negative | Negative | Negative | Female | 34 | - | 2.84 | 290 | 148 | 342 | - | Normal |
| 7 | Positive | Negative | Negative | Female | 63 | PV | 12.74 | 575 | 132 | 12.63 | Splenomegaly, (43×150) | Normal |
| 8 | Positive | Negative | Negative | Male | 65 | PV | 10.33 | 287 | 195 | 975.5 | Splenomegaly, (44×112) | Normal |
| 9 | Positive | Negative | Negative | Female | 83 | ET | 11.09 | 377 | 100 | 173 | Splenomegaly, (99×269) | The two cells have abnormal structures on chromosomes 7 and 18 respectively, but do not form clones. |
| 10 | Positive | Negative | Negative | Male | 71 | ET | 21.43 | 797 | 142 | 130 | Splenomegaly, (53×180) | One cell showed a polyploid karyotype, with structural abnormalities on chromosome 19. |
| 11 | Positive | Negative | Negative | Male | 64 | PMF | 44.78 | 160 | 118 | 557 | Splenomegaly, (53×190) | Normal |
| 12 | Positive | Negative | Negative | Female | 59 | PMF | 10.8 | 158 | 129 | 126 | Splenomegaly, (77×277) | 46,XX,add(3)(q12)[5]/46,XX[15] |
