# Supplementary material for: Dnmt3a is downregulated by Stat5a and mediates G0/G1 arrest by suppressing the miR-17-5p/Cdkn1a axis in Jak2V617F cells
Source: BMC Cancer. 2021 Nov 13;21:1213. doi: 10.1186/s12885-021-08915-0 (PMC8590245; doi:10.1186/s12885-021-08915-0)
Supplement: Supplementary file 2 — Additional file 2: Table S2. Primer sequences of different promoter regions of Dnmt3a (− 2860 bp to + 140 bp) [file 12885_2021_8915_MOESM2_ESM.docx]

**Table S2**

Primer sequences of different promoter regions of Dnmt3a (-2860 bp to +140 bp)

| Name | Primer | Sequence (5’-3’) | Product size |
| --- | --- | --- | --- |
| P1 | Forward | tctgtgtagccttggttgtcc | 2815bp |
|  | Reverse | agcttgatggcagagtgctt |  |
| P2 | Forward | ctatcgtaaggtggcacccc | 2083bp |
|  | Reverse | agcttgatggcagagtgctt |  |
| P3 | Forward | tcaaagaggtcgtgacccac | 1432bp |
|  | Reverse | agcttgatggcagagtgctt |  |
| P4 | Forward | tagccctggctgtattggaac | 689bp |
|  | Reverse | agcttgatggcagagtgctt |  |
| P^3/4^ | Forward | tctgtgtagccttggttgtcc | 2147bp |
|  | Reverse | gttccaatacagccagggcta |  |
| Region 1 | Forward | tctgtgtagccttggttgtcc | 752bp |
|  | Reverse | ggggtgccaccttacgatag |  |
| Region 2 | Forward | ctatcgtaaggtggcacccc | 671bp |
|  | Reverse | gtgggtcacgacctctttga |  |
| Region 3 | Forward | tcaaagaggtcgtgacccac | 764bp |
|  | Reverse | gttccaatacagccagggcta |  |
