# Supplementary figures and images for: Dnmt3a is downregulated by Stat5a and mediates G0/G1 arrest by suppressing the miR-17-5p/Cdkn1a axis in Jak2V617F cells
Source: BMC Cancer. 2021 Nov 13;21:1213. doi: 10.1186/s12885-021-08915-0 (PMC8590245; doi:10.1186/s12885-021-08915-0)

Supplementary Figure 1 (uncropped images of Figure 1A)

Jak2

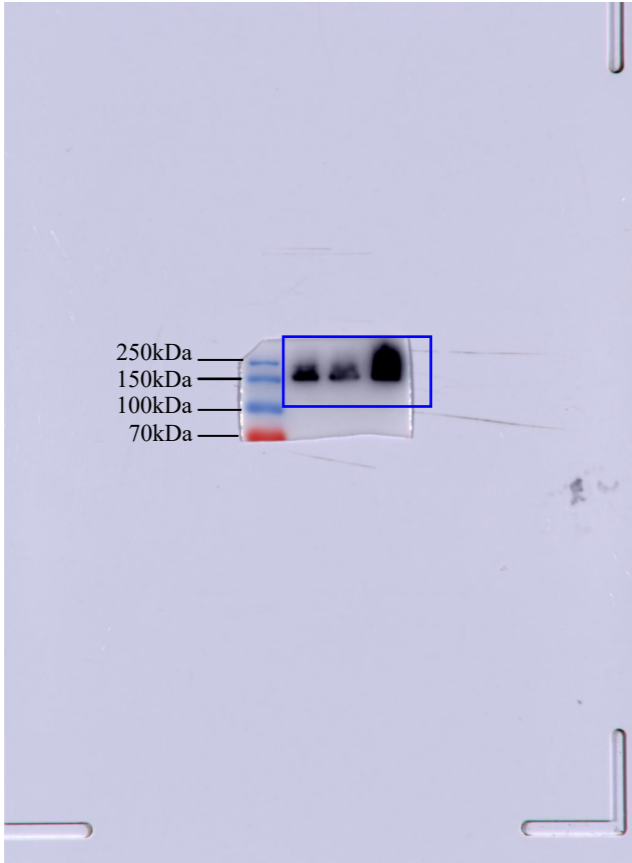

p-Jak2

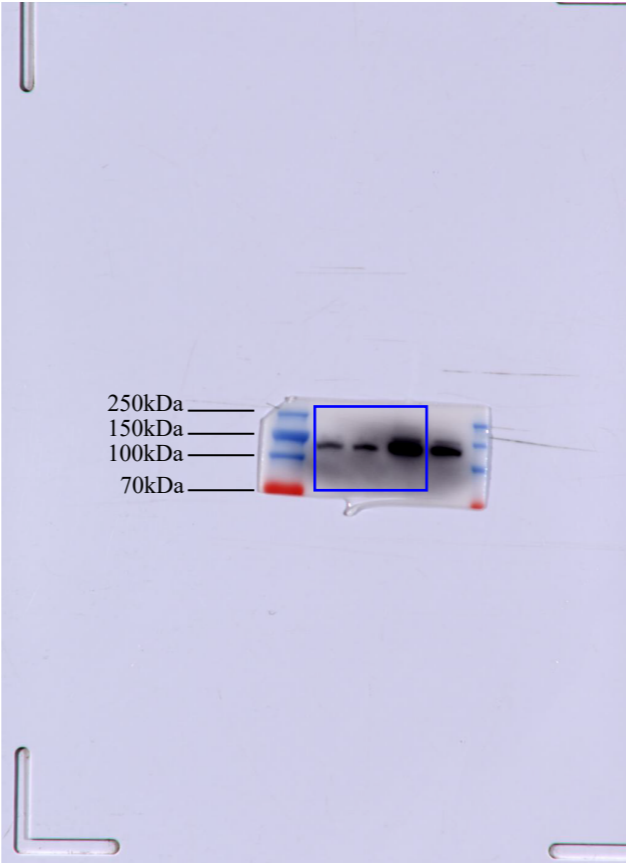

Stat5a

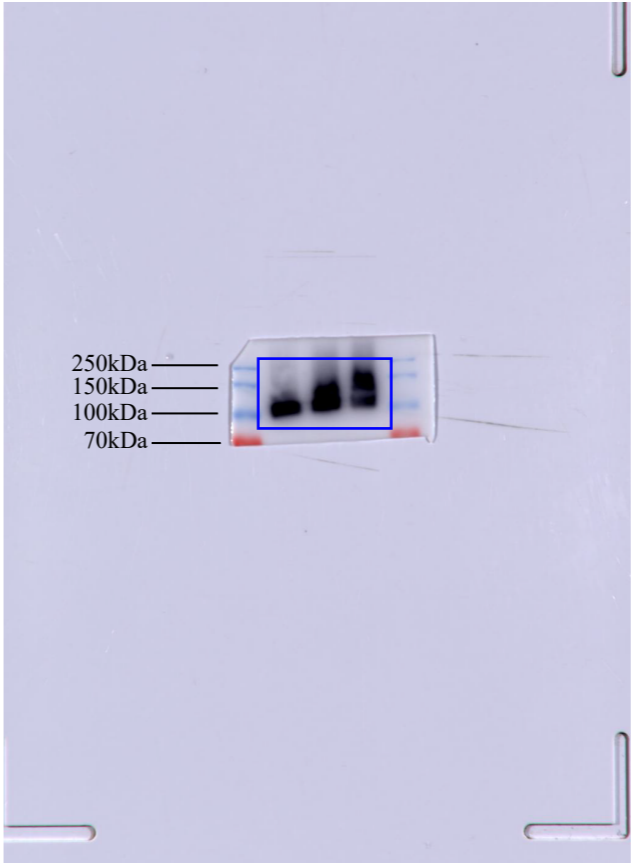

p-Stat5a

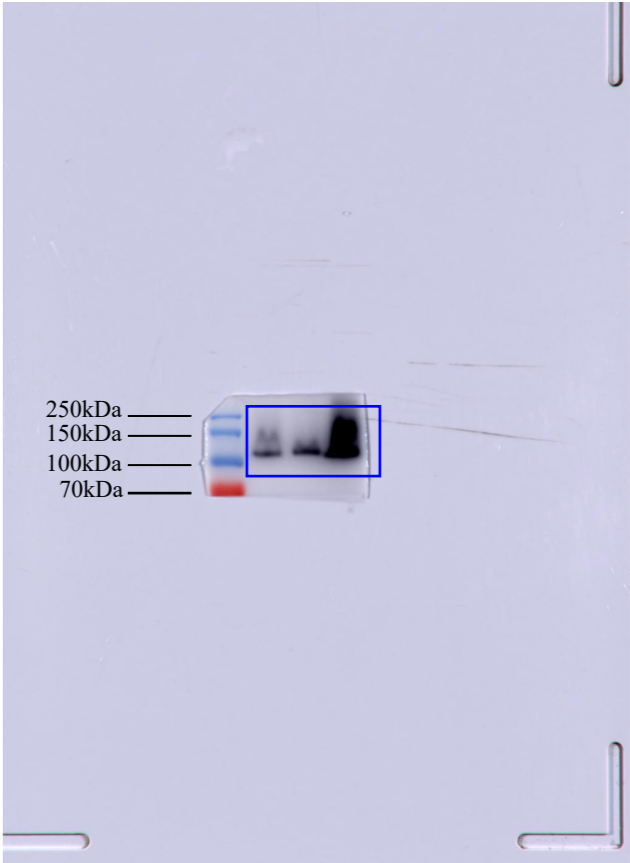

Dnmt3a

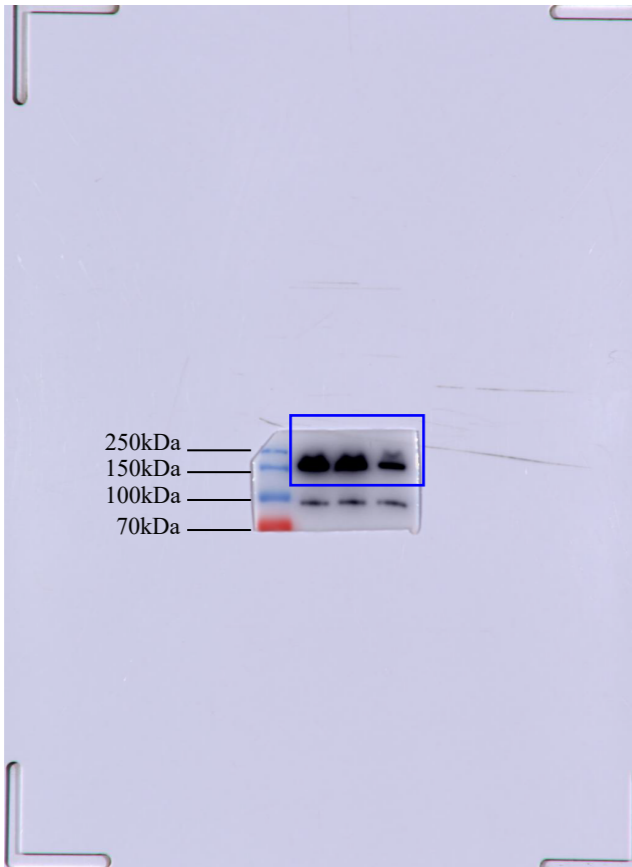

$\beta$ -actin

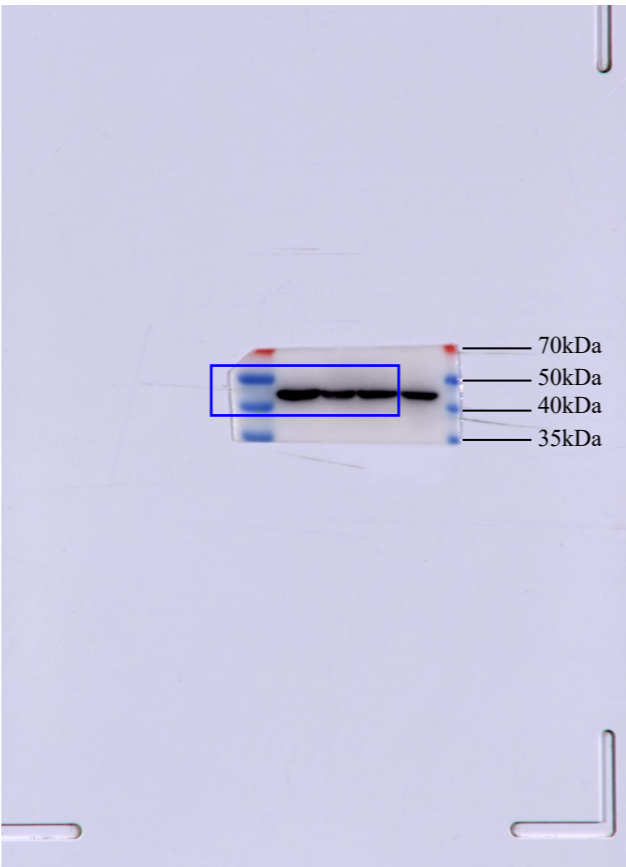

Supplement: Supplementary file 3 — Additional file 3: Fig. S1. Uncropped images of Fig. 1A [file 12885_2021_8915_MOESM3_ESM.pdf]

**Supplementary Figure 2 (uncropped images of Figure 1C)**

p-Stat5a

Dnmt3a

$\beta$ -actin

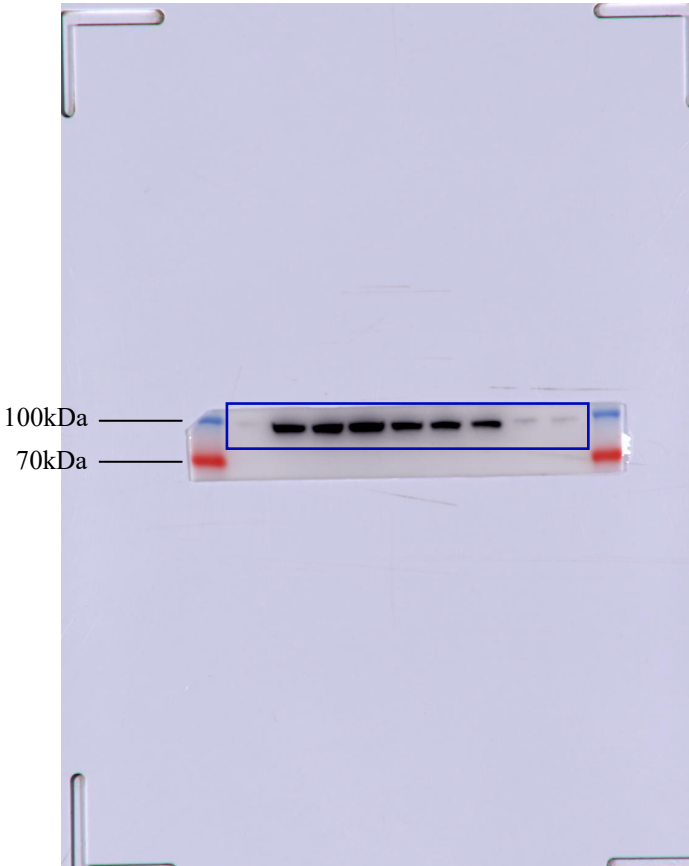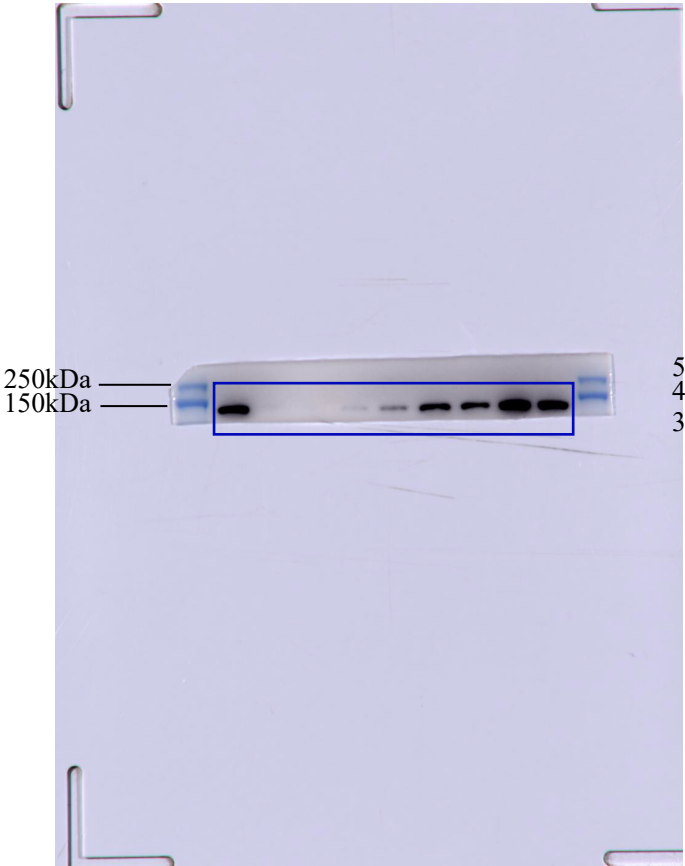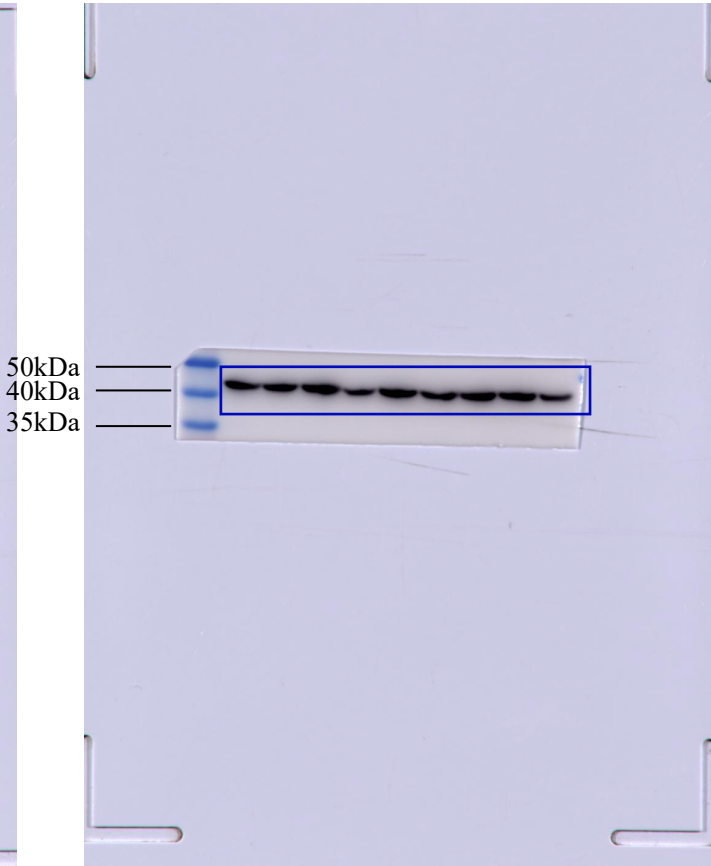

Supplement: Supplementary file 4 — Additional file 4: Fig. S2. Uncropped images of Fig. 1C [file 12885_2021_8915_MOESM4_ESM.pdf]

## Supplementary Figure 3 (uncropped images of Figure 1D)

p-Stat5a

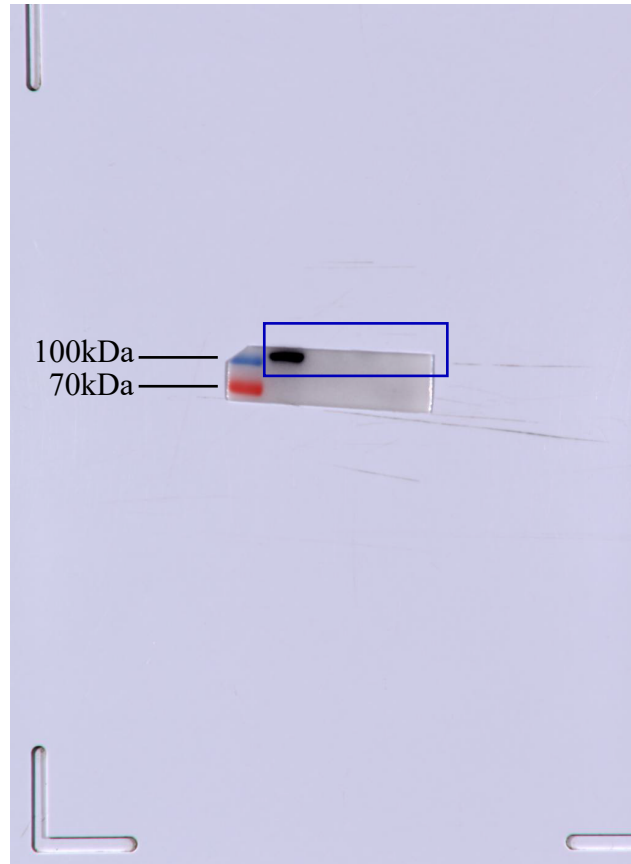

Dnmt3a

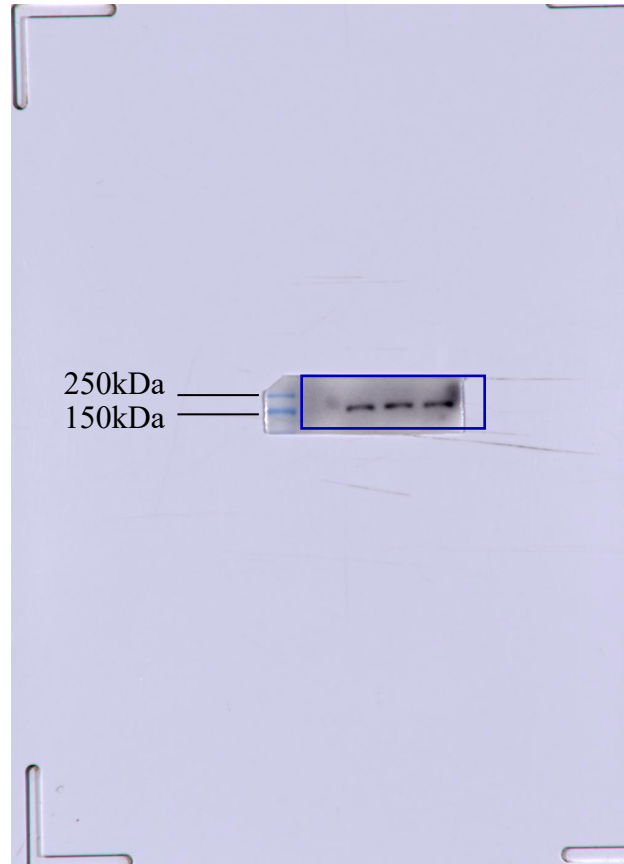

$\beta$ -actin

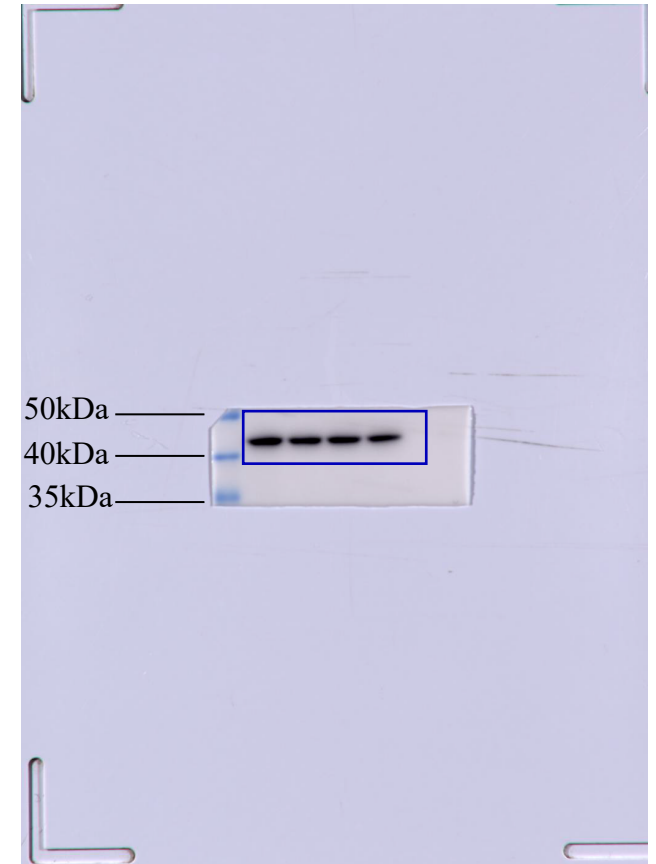

Supplement: Supplementary file 5 — Additional file 5: Fig. S3. Uncropped images of Fig. 1D [file 12885_2021_8915_MOESM5_ESM.pdf]

Supplementary Figure 4 (uncropped images of Figure 1E )

Stat5a

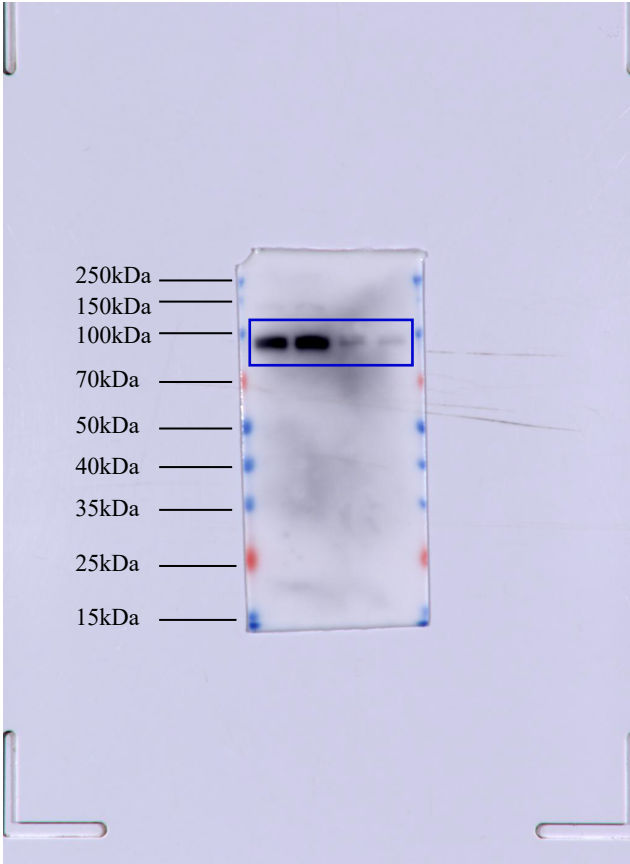

p-Stat5a

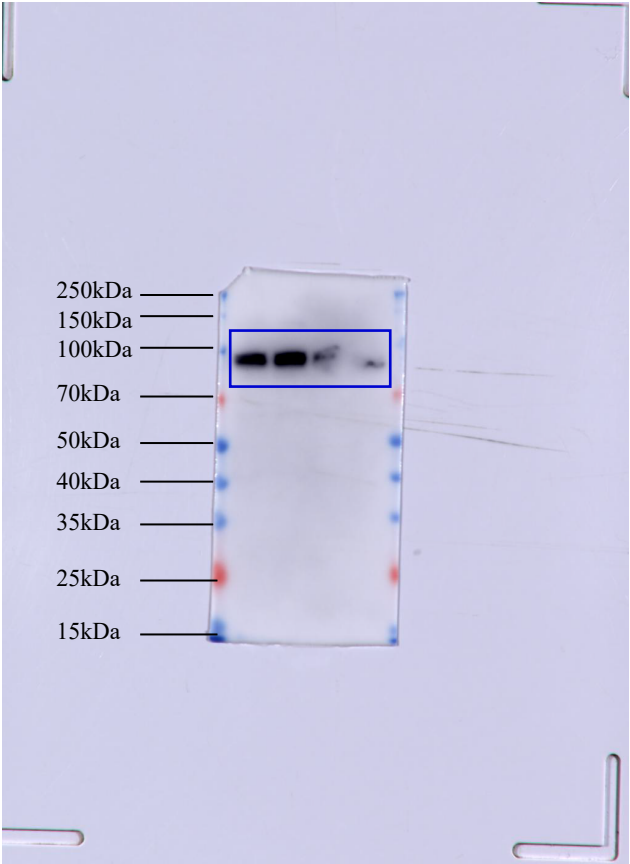

Dnmt3a

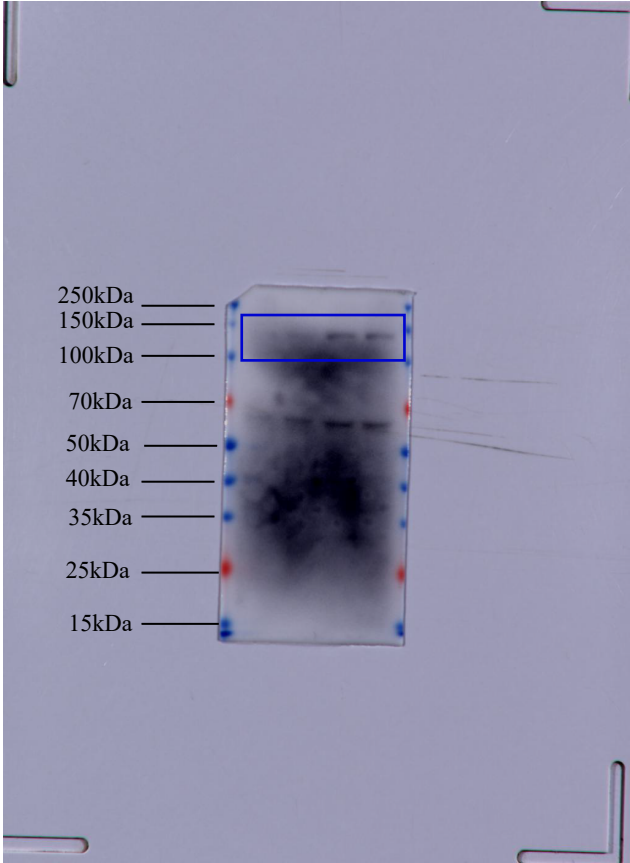

$\beta$ -actin

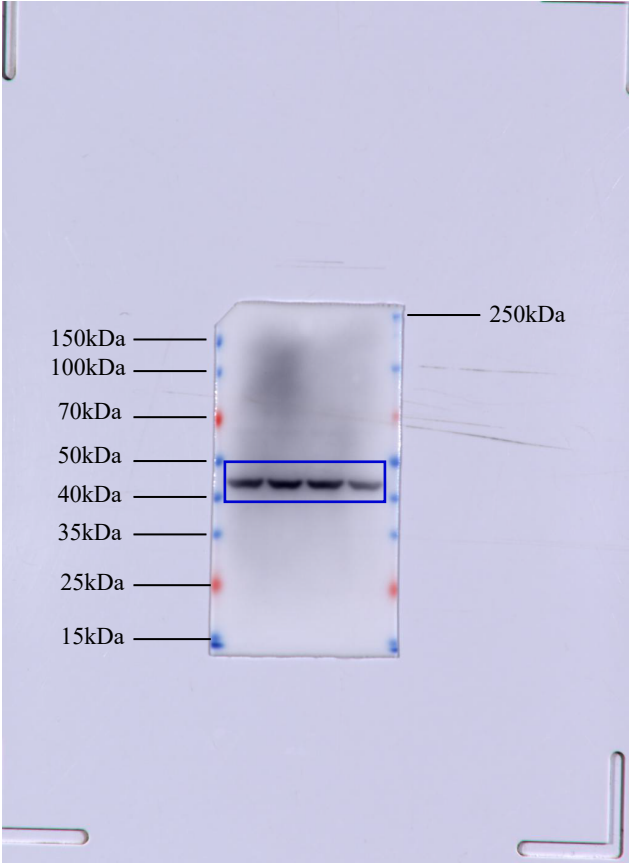

Supplement: Supplementary file 6 — Additional file 6: Fig. S4. Uncropped images of Fig. 1E [file 12885_2021_8915_MOESM6_ESM.pdf]

## Supplementary Figure 5 (uncropped images of Figure 2A)

p-STAT5a

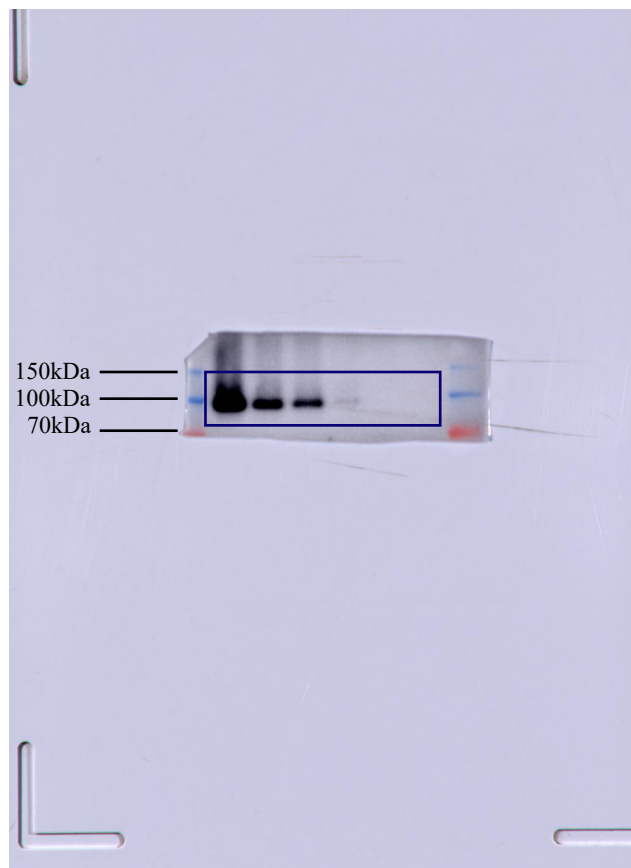

DNMT3a

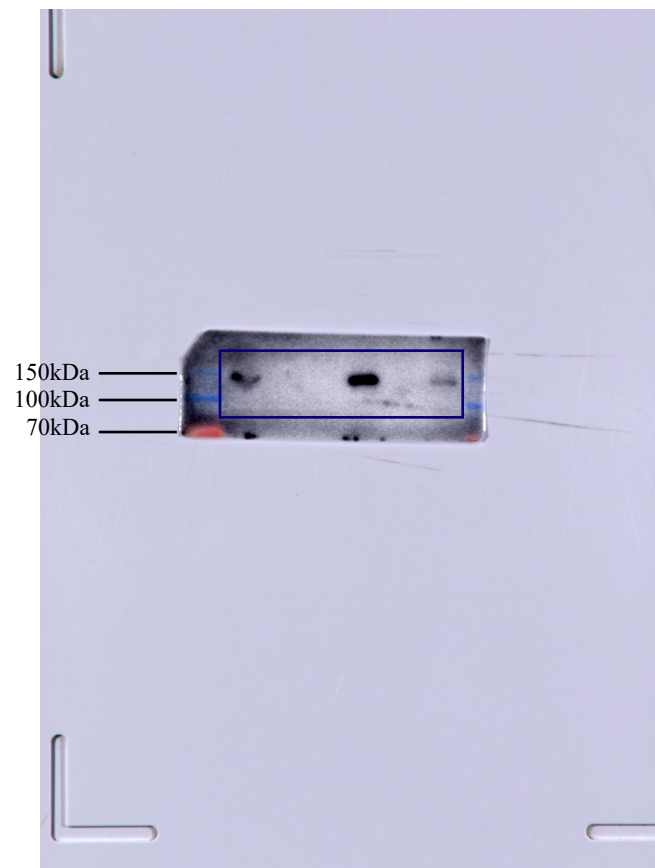

$\beta$ -actin

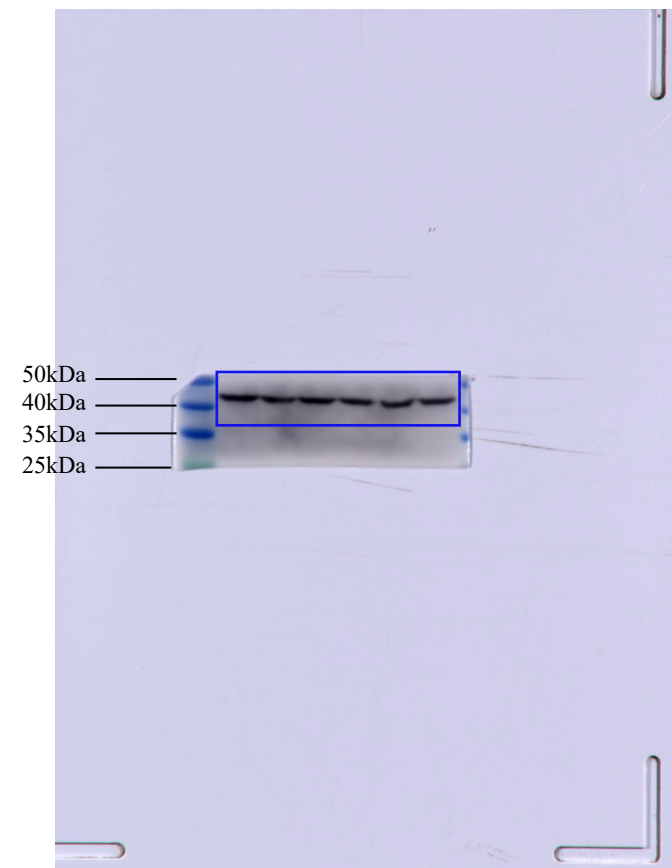

Supplement: Supplementary file 7 — Additional file 7: Fig. S5. Uncropped images of Fig. 2A [file 12885_2021_8915_MOESM7_ESM.pdf]

## Supplementary Figure 6 (uncropped images of Figure 2B)

p-Stat5a

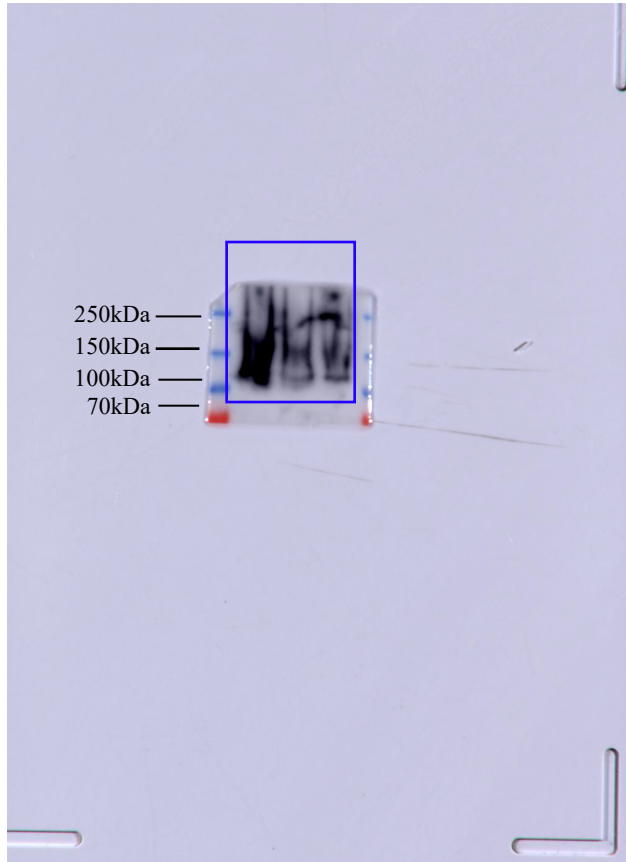

Dnmt3a

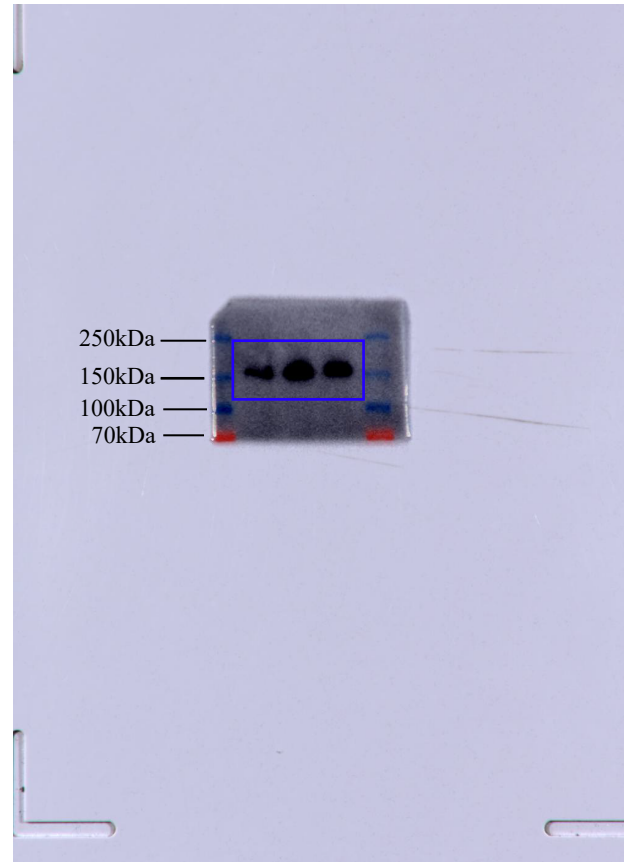

$\beta$ -actin

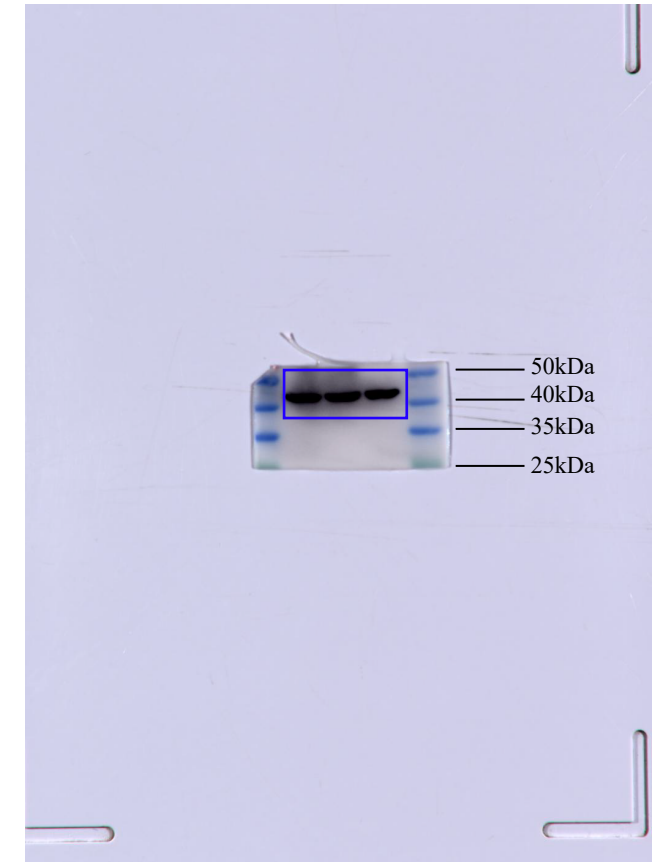

Supplement: Supplementary file 8 — Additional file 8: Fig. S6. Uncropped images of Fig. 2B [file 12885_2021_8915_MOESM8_ESM.pdf]

Supplementary Figure 7 (uncropped images of Figure 2C)

Stat5a

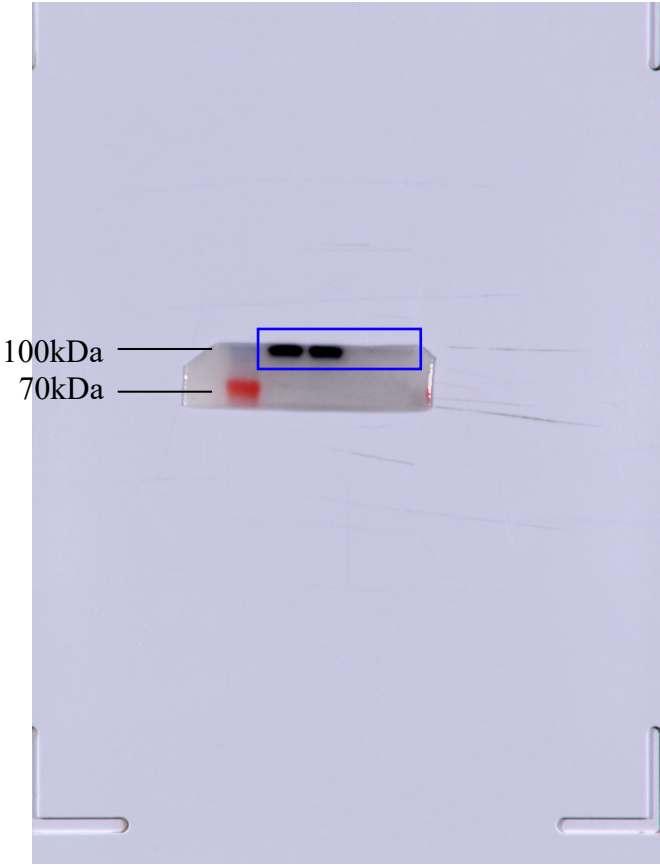

Dnmt3a

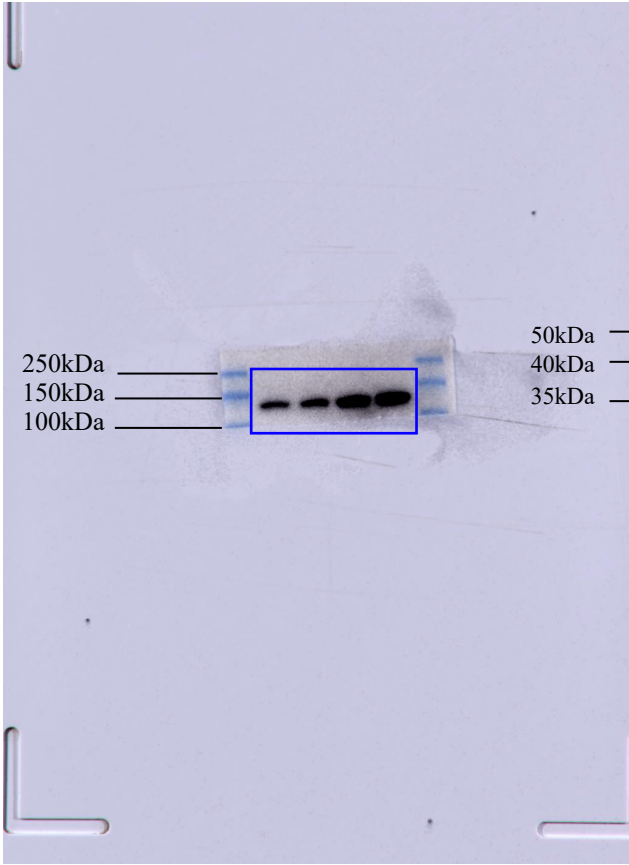

$\beta$ -actin

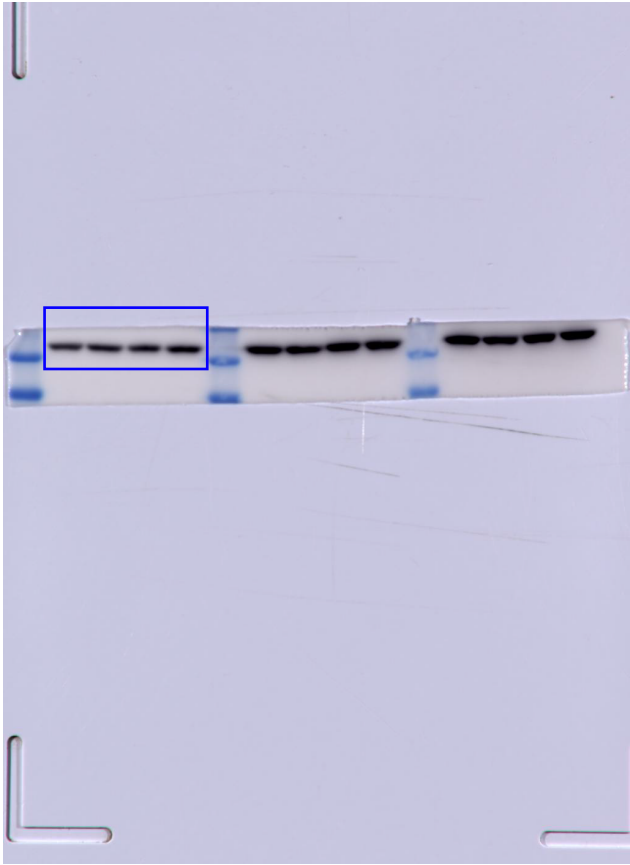

Supplement: Supplementary file 9 — Additional file 9: Fig. S7. Uncropped images of Fig. 2C [file 12885_2021_8915_MOESM9_ESM.pdf]

Supplementary Figure 8 (uncropped images of Figure 2D)

Stat5a

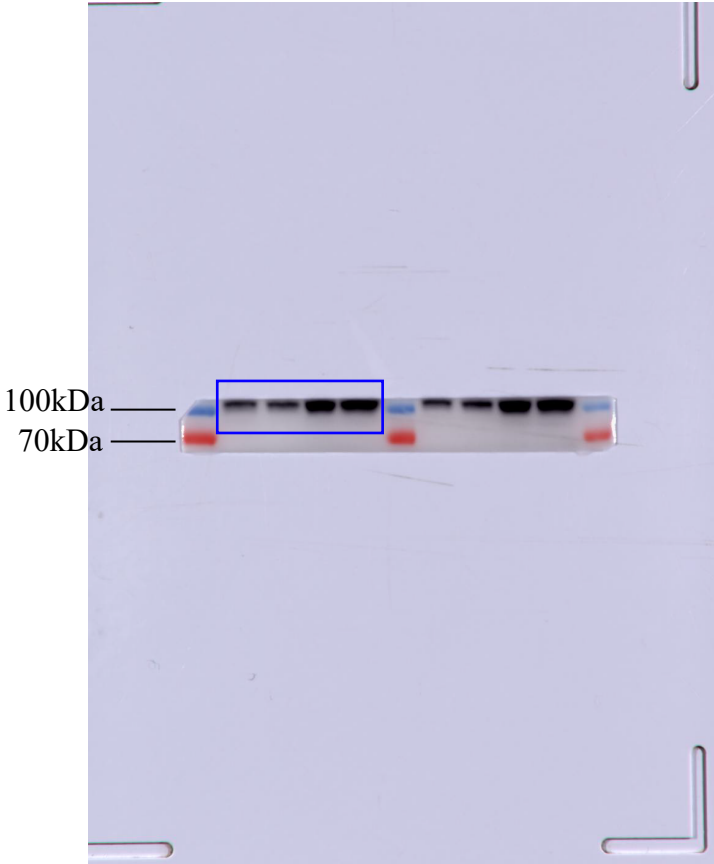

Dnmt3a

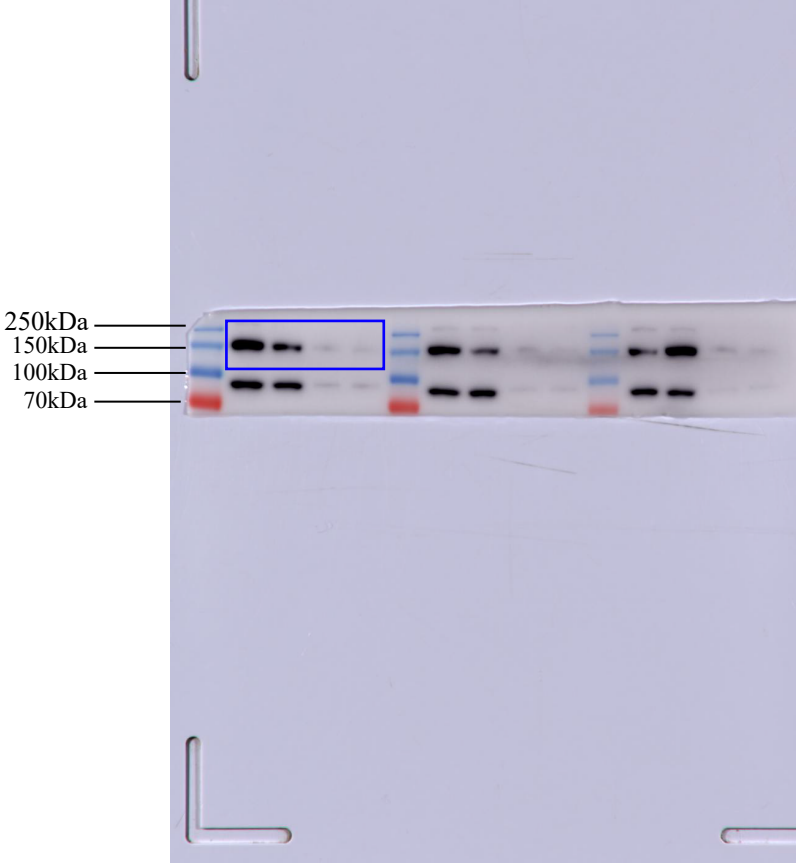

$\beta$ -actin

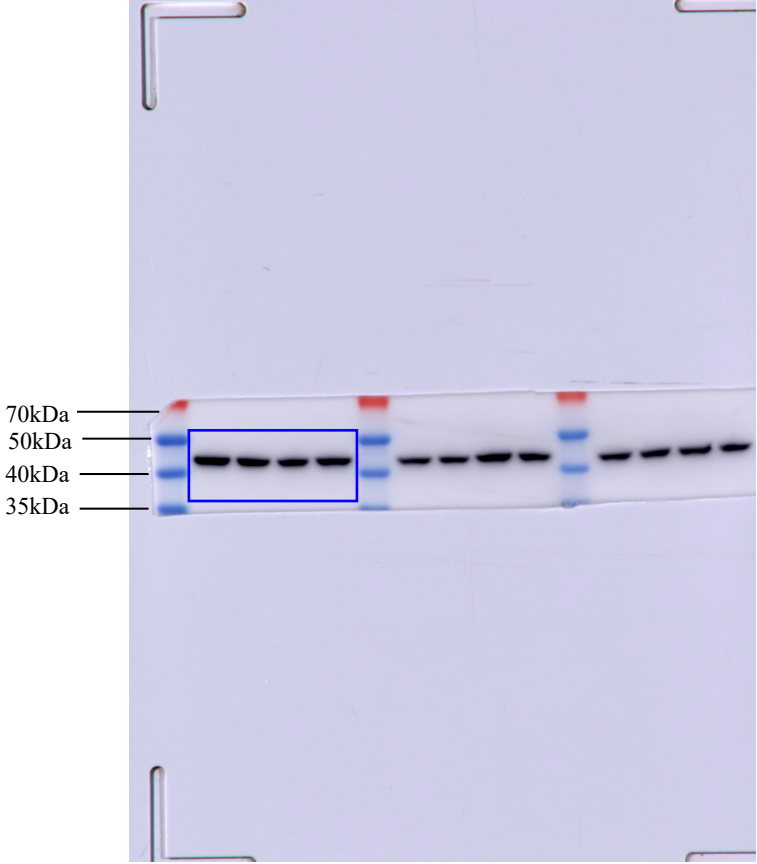

Supplement: Supplementary file 10 — Additional file 10: Fig. S8. Uncropped images of Fig. 2D [file 12885_2021_8915_MOESM10_ESM.pdf]

Supplementary Figure 9 (uncropped images of Figure 2E)

Stat5a

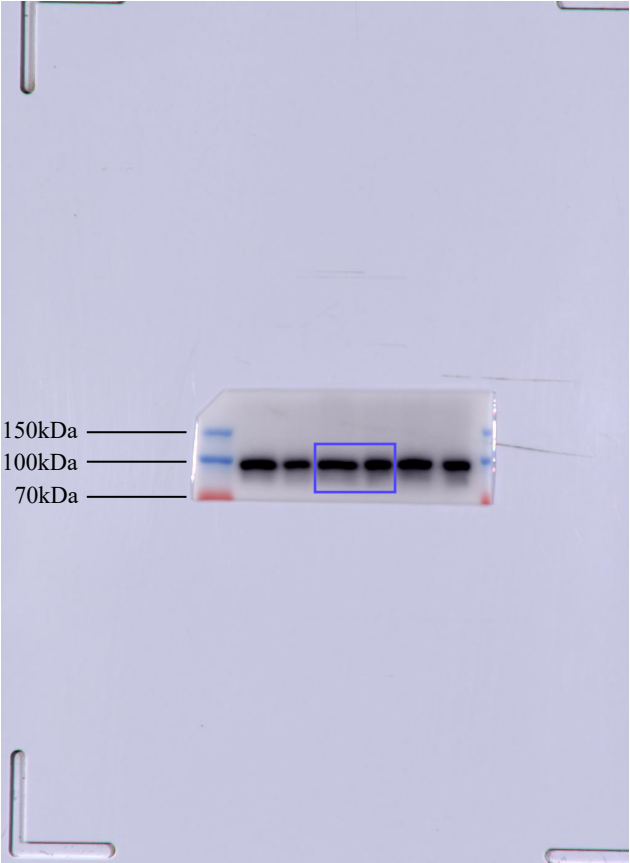

Dnmt3a

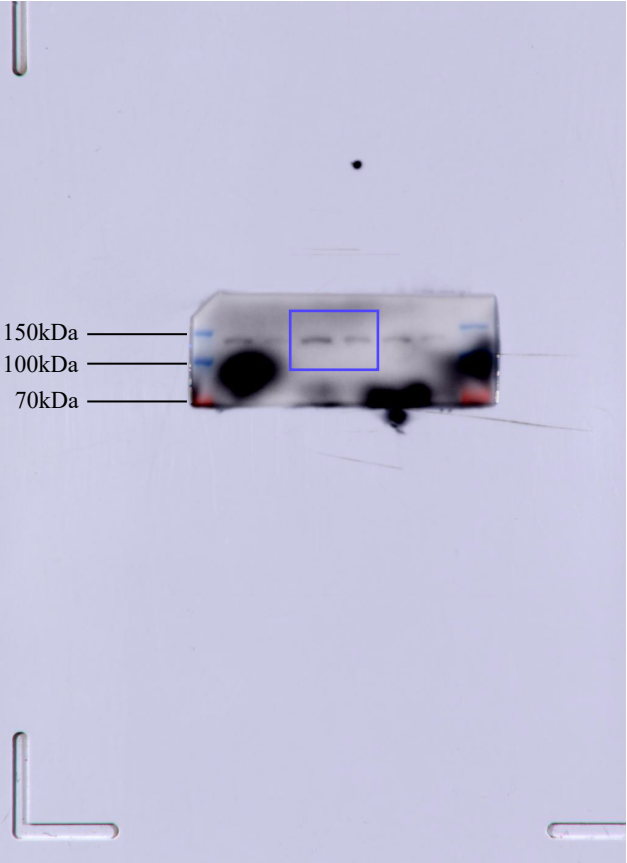

$\beta$ -actin

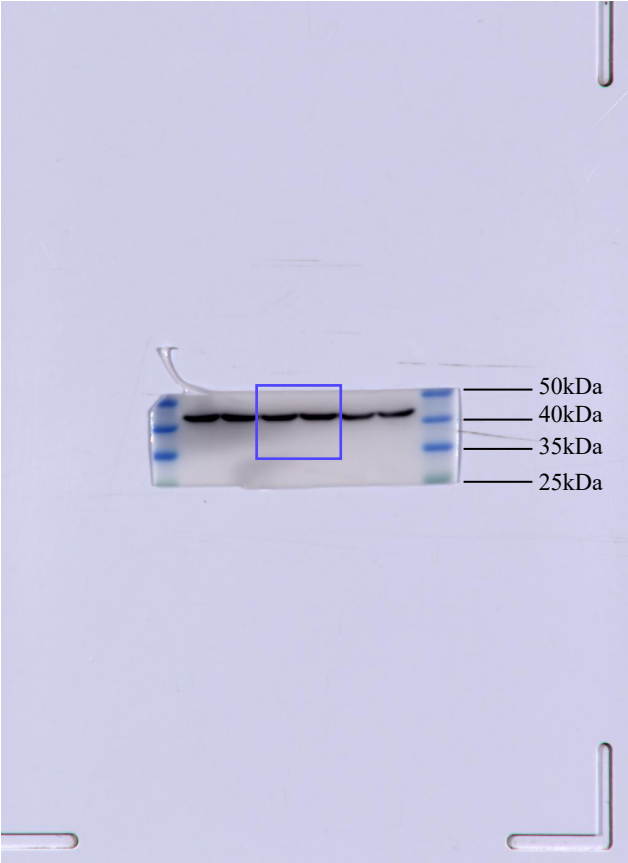

Supplement: Supplementary file 11 — Additional file 11: Fig. S9. Uncropped images of Fig. 2E [file 12885_2021_8915_MOESM11_ESM.pdf]

## Supplementary Figure 10 (uncropped images of Figure 2F)

Stat5a

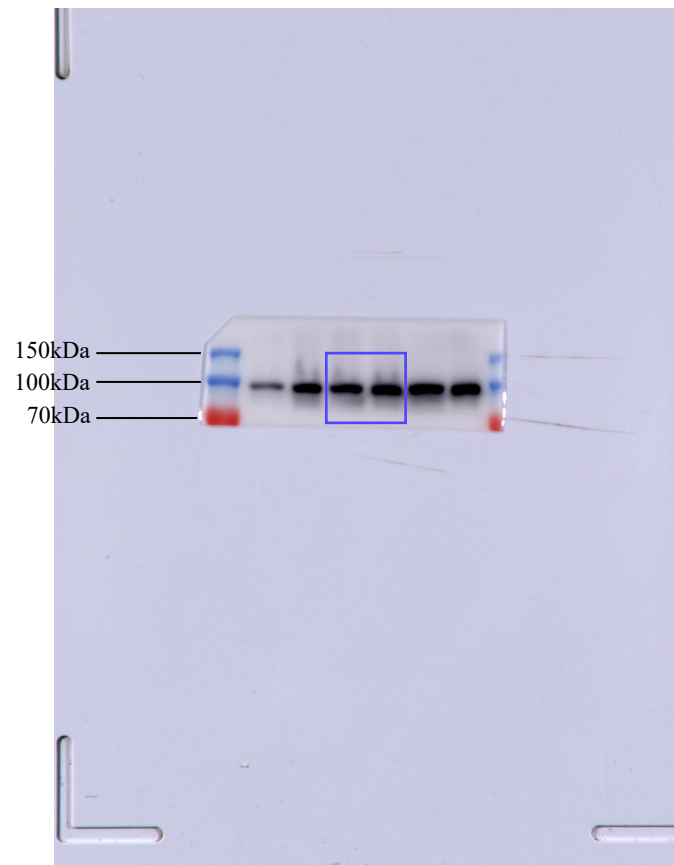

Dnmt3a

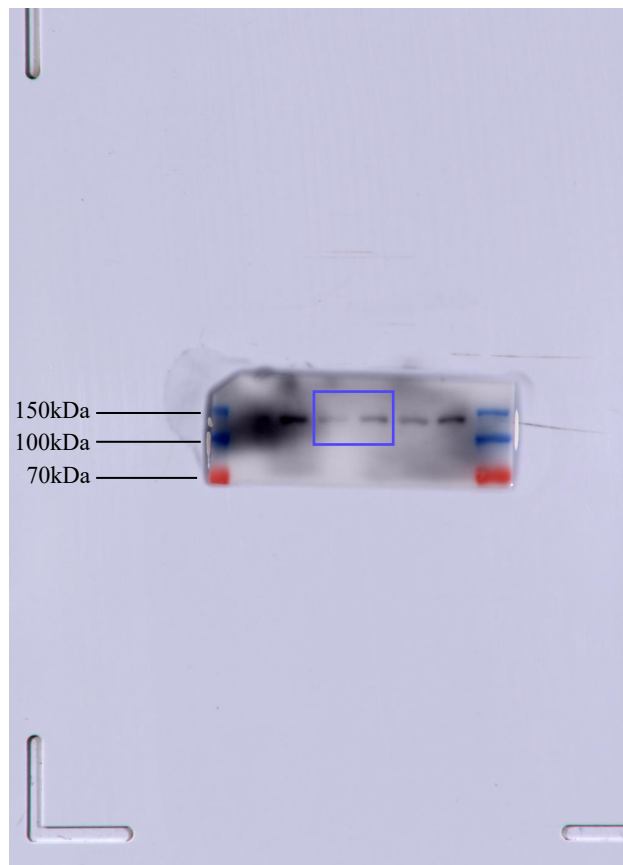

$\beta$ -actin

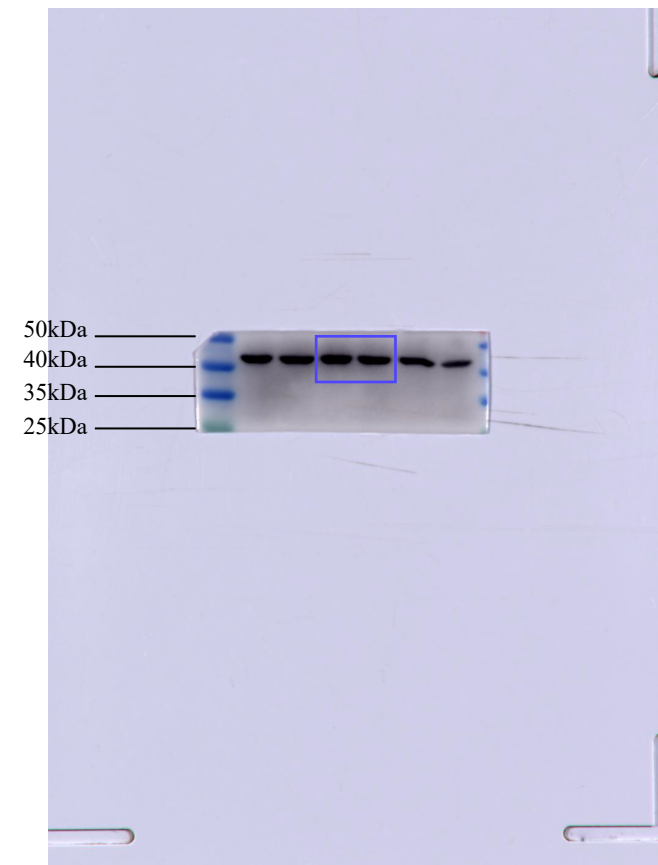

Supplement: Supplementary file 12 — Additional file 12: Fig. S10. Uncropped images of Fig. 2F [file 12885_2021_8915_MOESM12_ESM.pdf]

## Supplementary Figure 11 (uncropped images of Figure 2G)

Stat5a

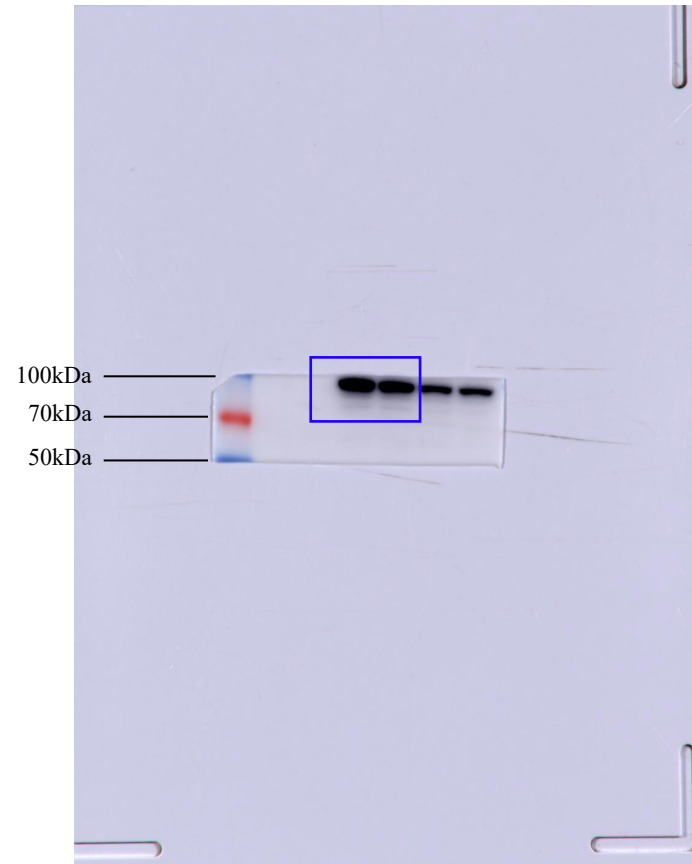

Dnmt3a

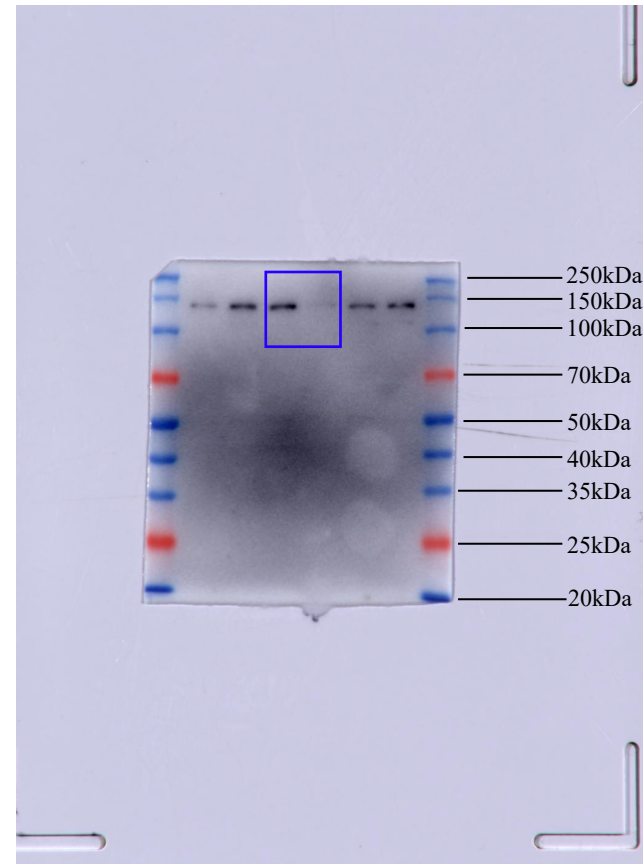

$\beta$ -actin

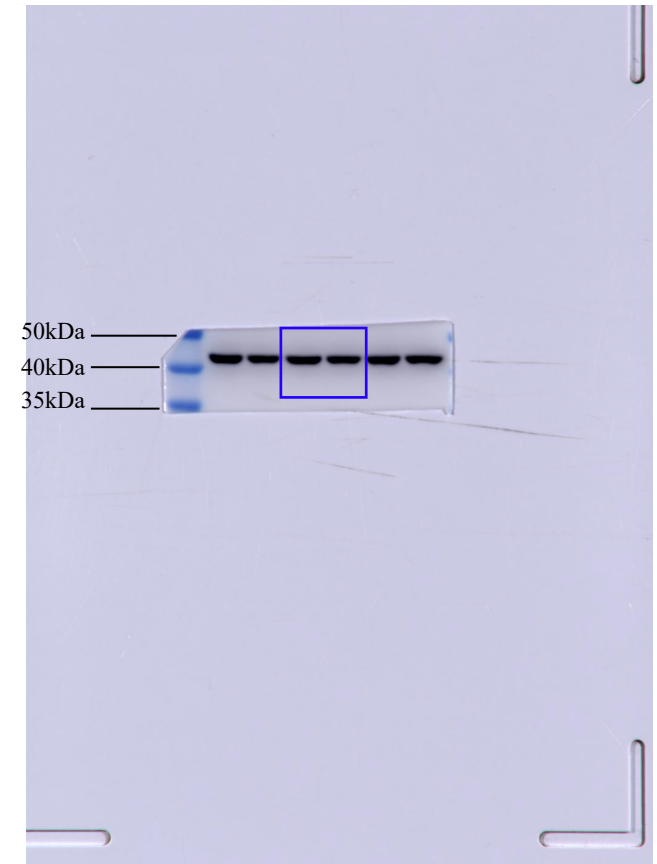

Supplement: Supplementary file 13 — Additional file 13: Fig. S11. Uncropped images of Fig. 2G [file 12885_2021_8915_MOESM13_ESM.pdf]

Supplementary Figure 12 (uncropped images of Figure 2H)

Stat5a

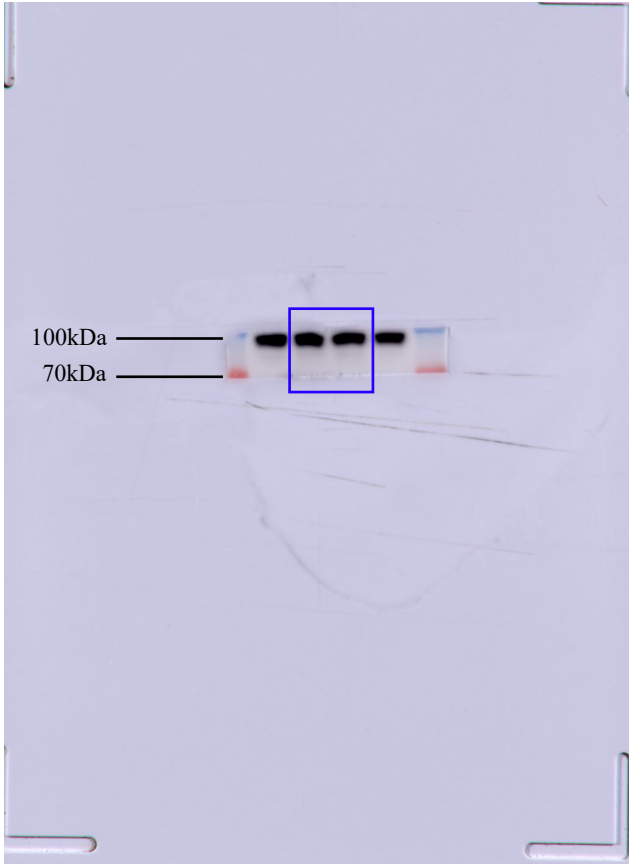

Dnmt3a

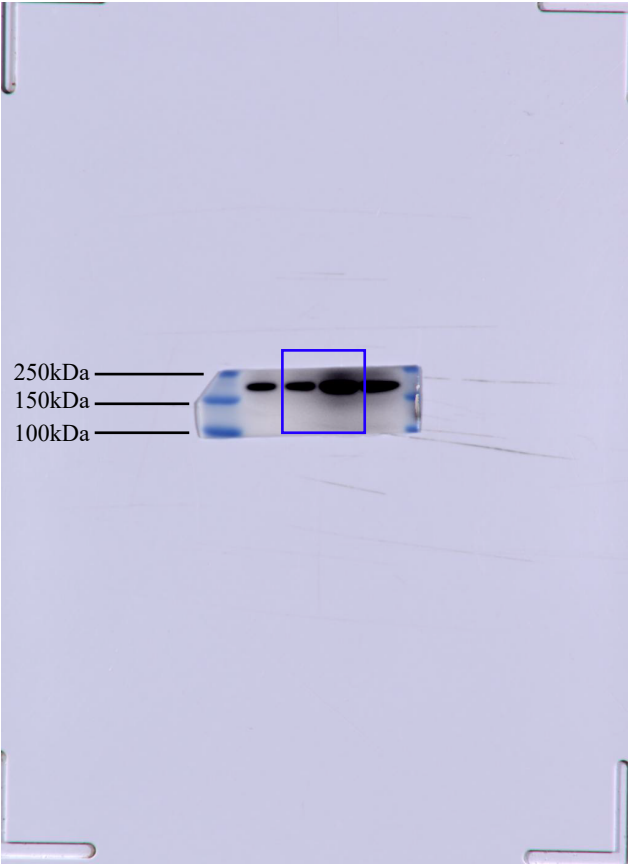

$\beta$ -actin

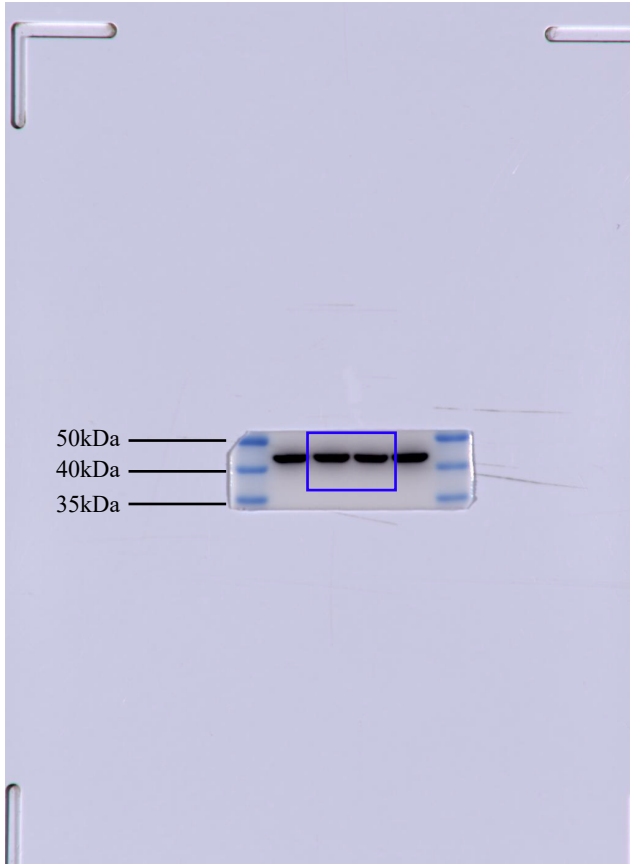

Supplement: Supplementary file 14 — Additional file 14: Fig. S12. Uncropped images of Fig. 2H [file 12885_2021_8915_MOESM14_ESM.pdf]

Supplementary Figure 13 (uncropped images of Figure 3E)

Stat5a

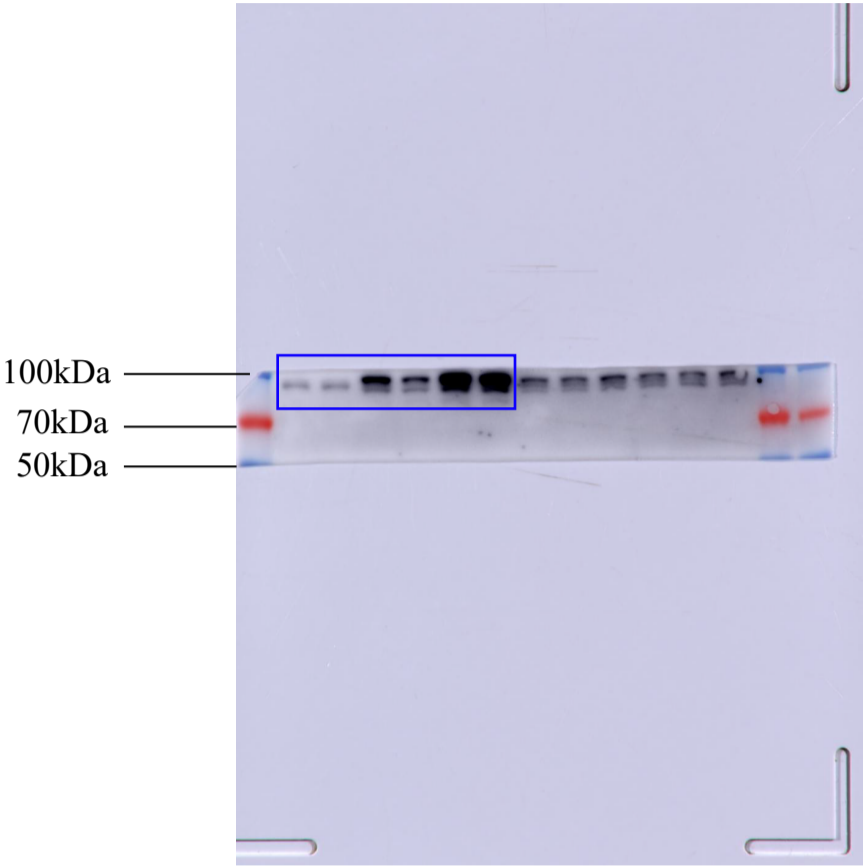

p-Stat5a

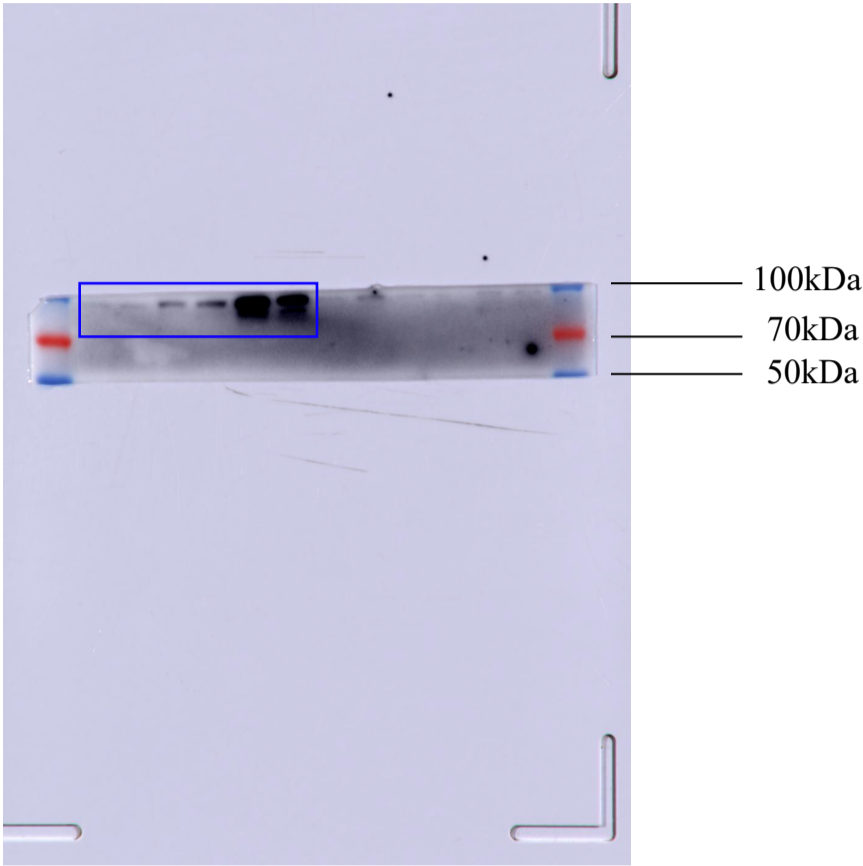

Dnmt3a

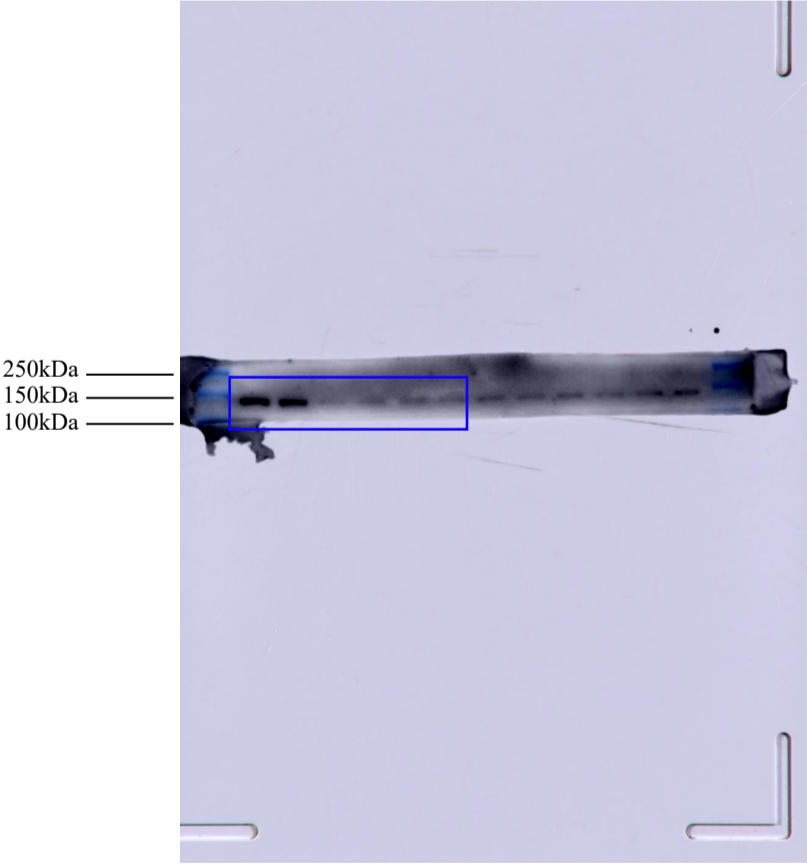

$\beta$ -actin

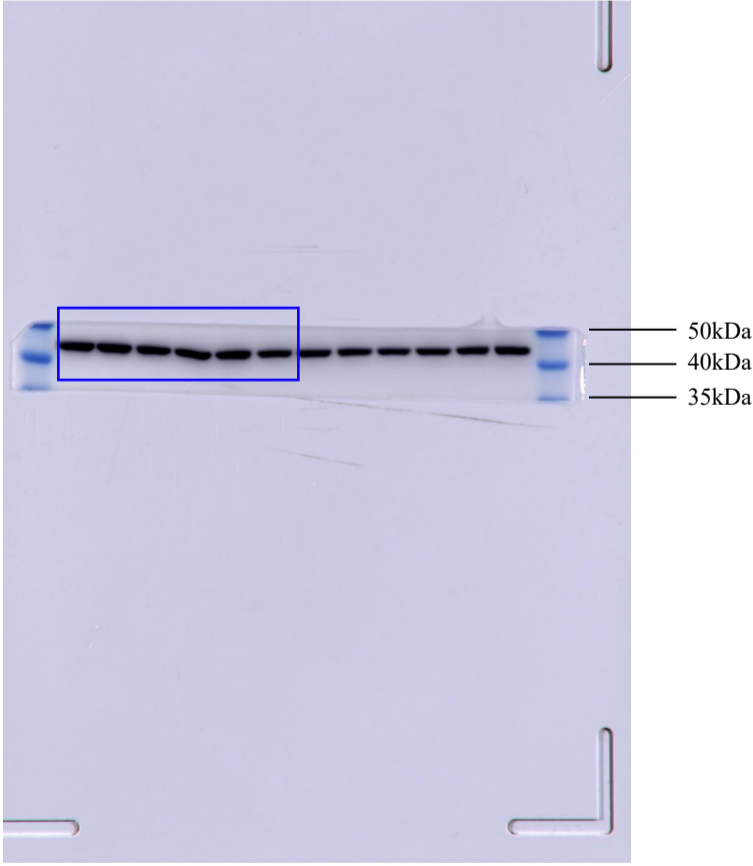

Supplement: Supplementary file 15 — Additional file 15: Fig. S13. Uncropped images of Fig. 3E [file 12885_2021_8915_MOESM15_ESM.pdf]

# Supplementary Figure 14 (uncropped images of Figure 4B)

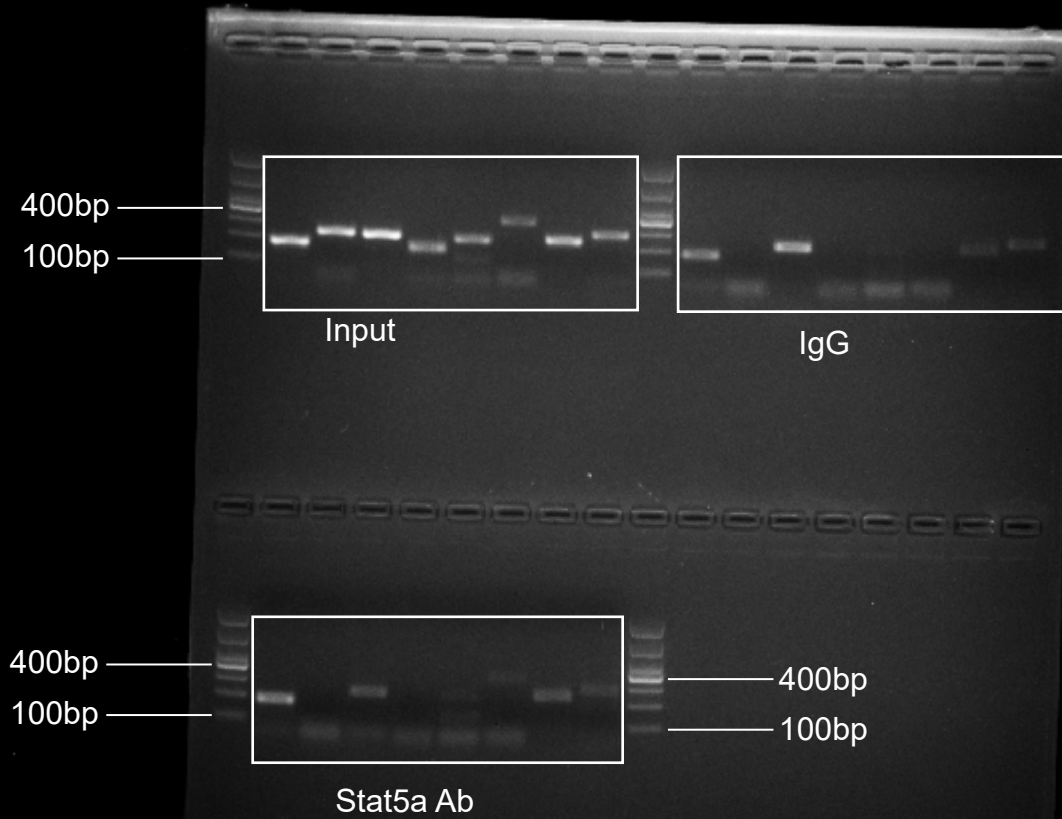

Supplement: Supplementary file 16 — Additional file 16: Fig. S14. Uncropped images of Fig. 4B [file 12885_2021_8915_MOESM16_ESM.pdf]

Supplementary Figure 15 (uncropped images of Figure 7A)

p-Stat5a

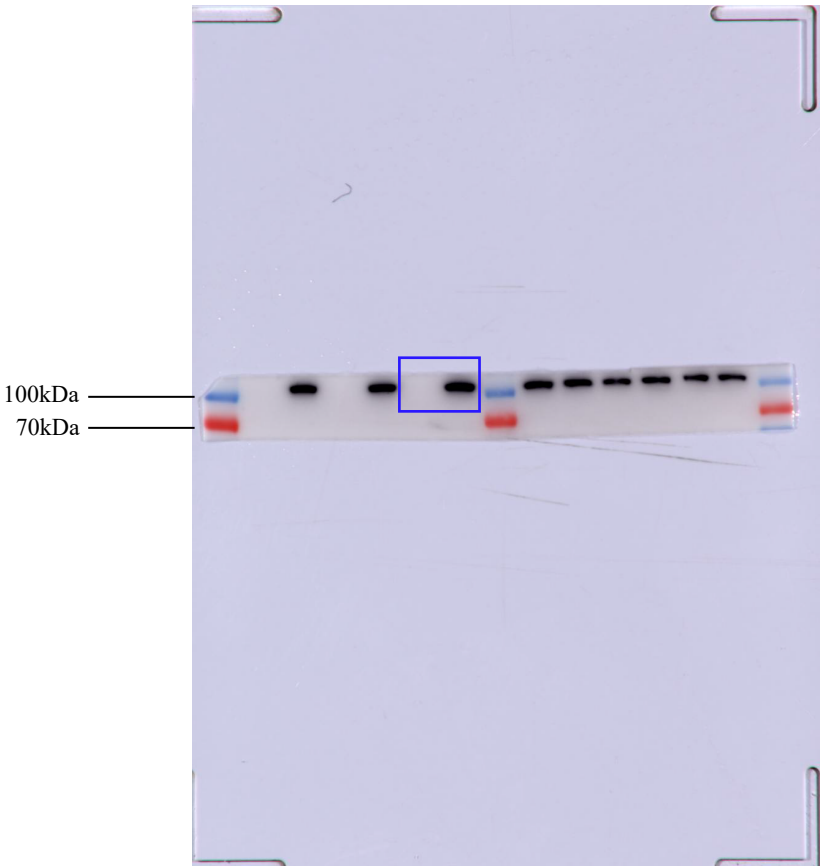

Dnmt3a

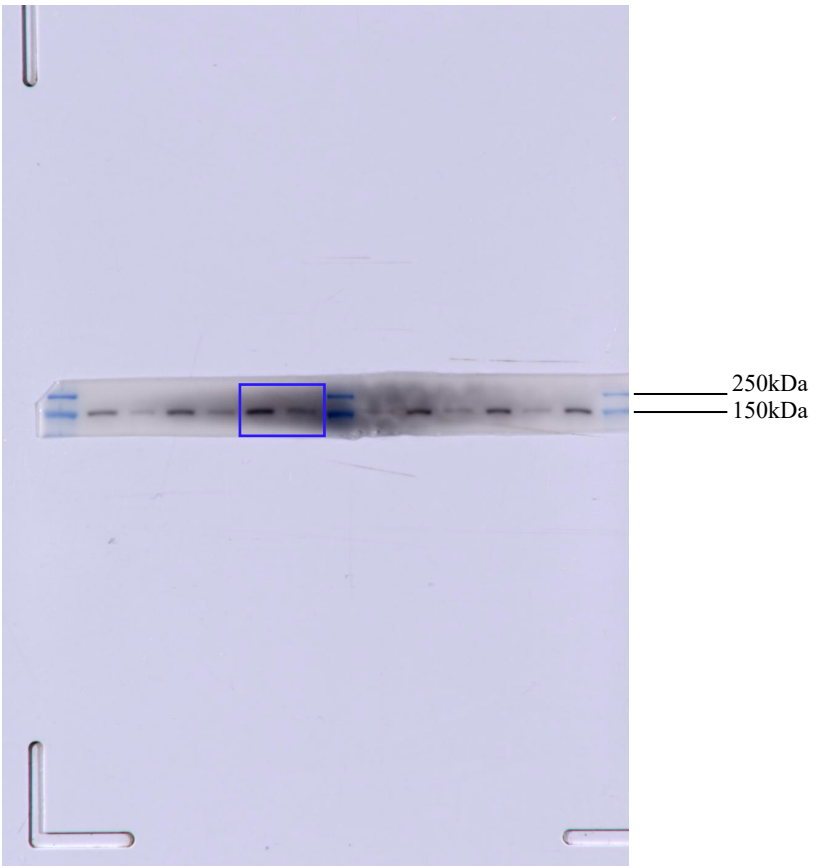

Cdkn1a

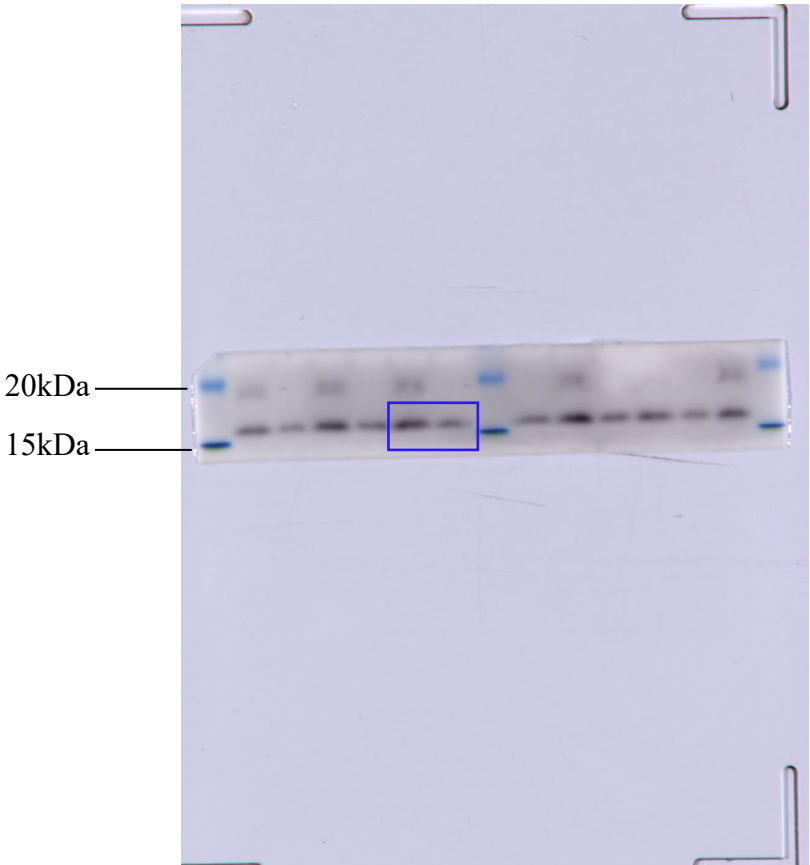

$\beta$ -actin

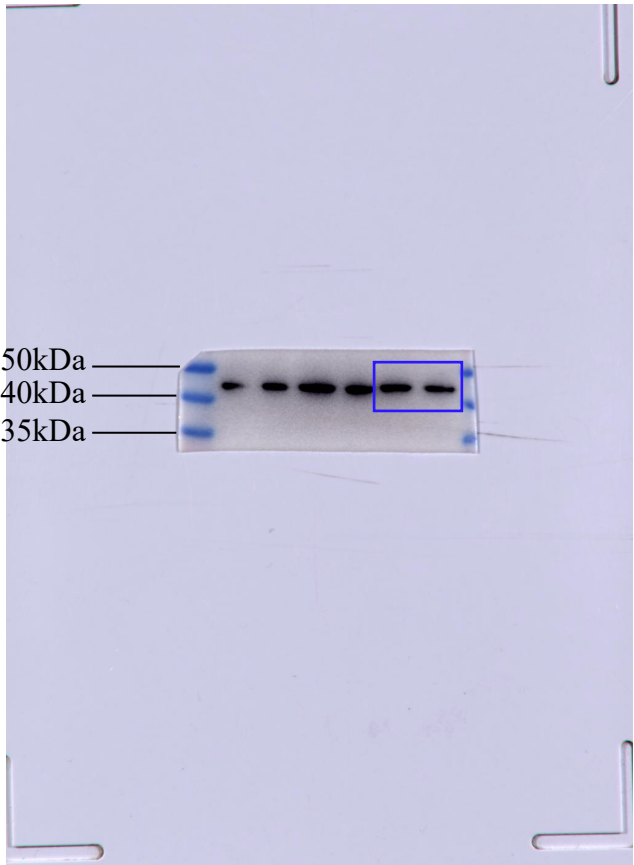

Supplement: Supplementary file 17 — Additional file 17: Fig. S15. Uncropped images of Fig. 7A [file 12885_2021_8915_MOESM17_ESM.pdf]

Supplementary Figure 16 (uncropped images of Figure 8A)

p-Stat5a

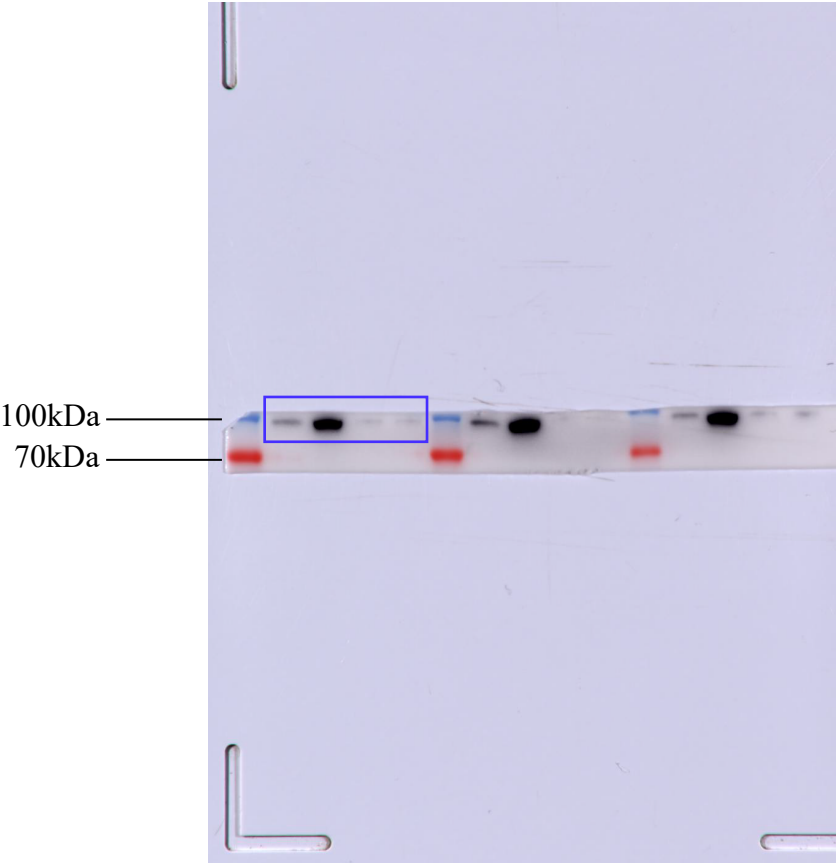

Dnmt3a

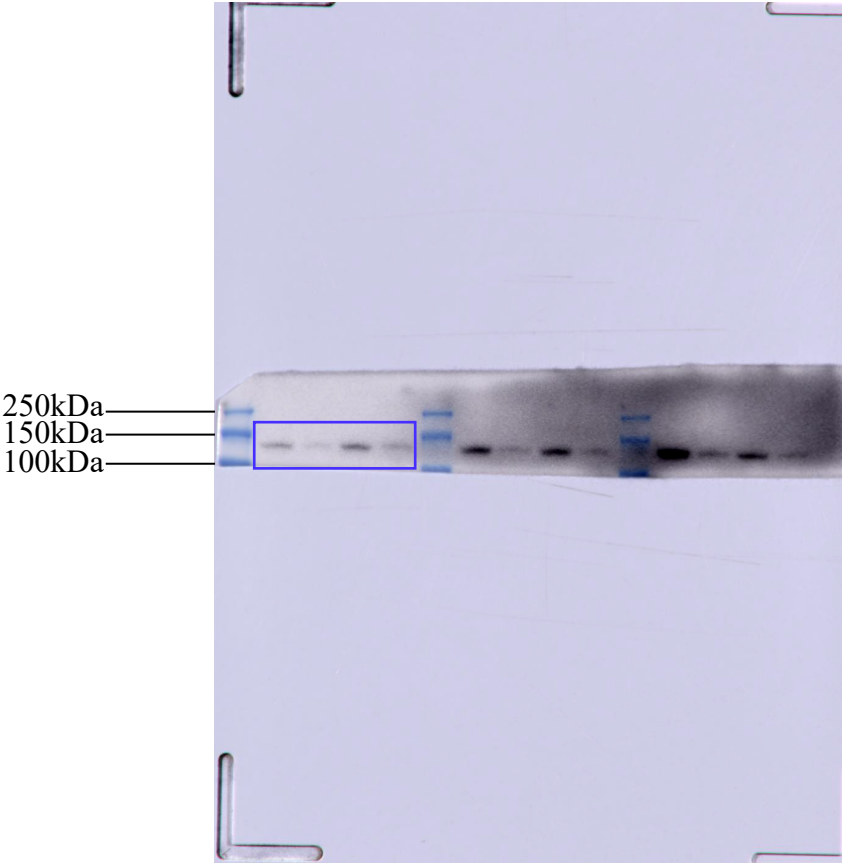

Cdkn1a

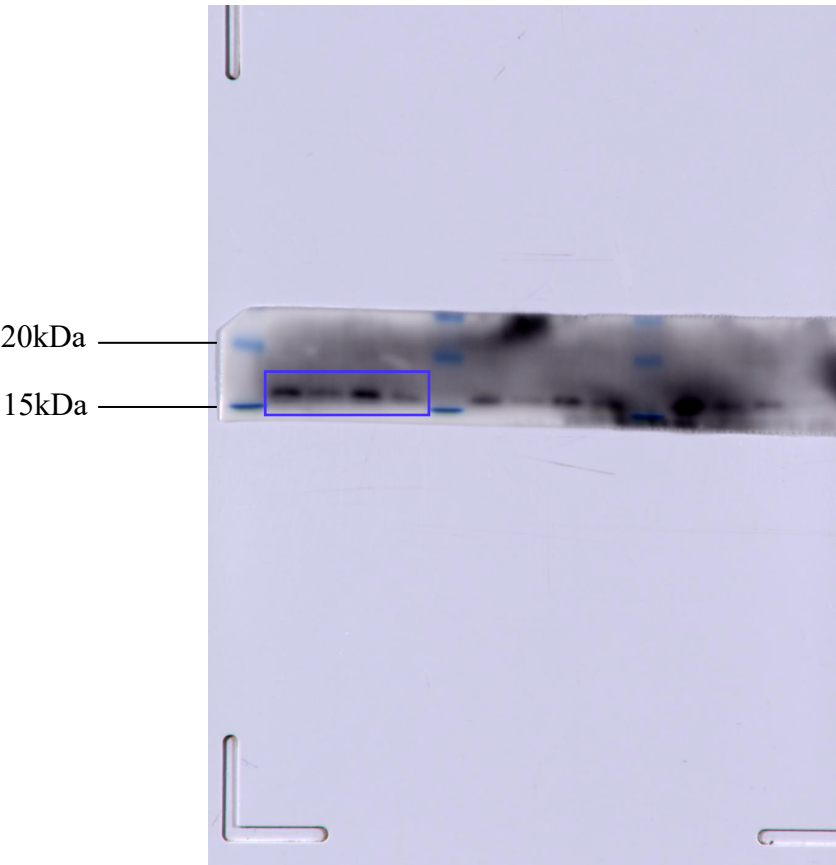

$\beta$ -actin

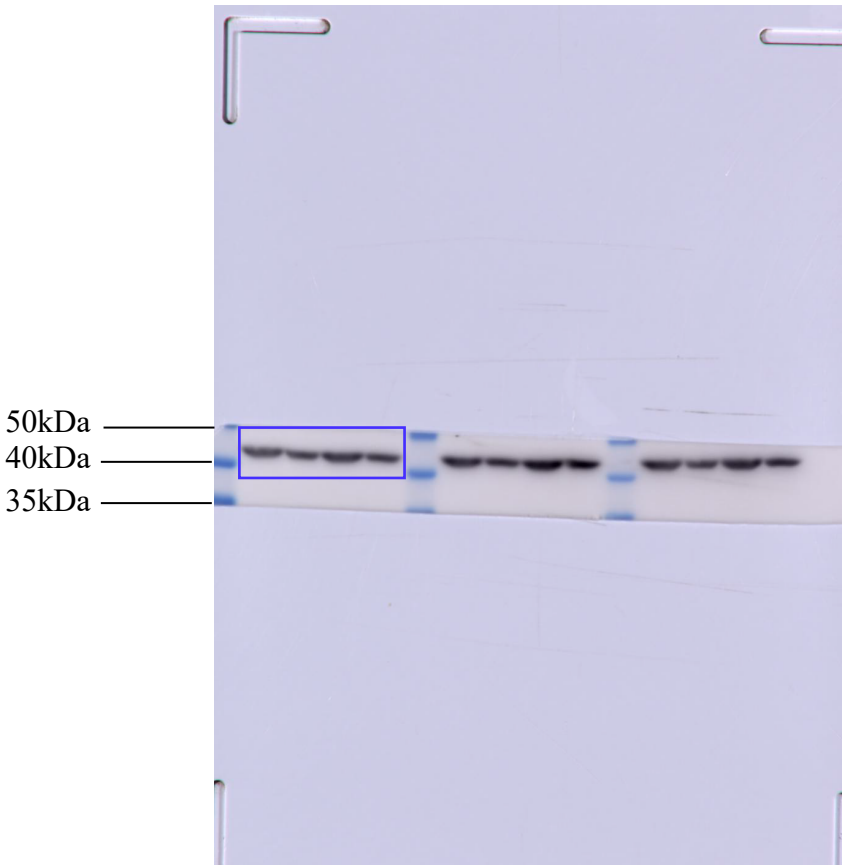

Supplement: Supplementary file 18 — Additional file 18: Fig. S16. Uncropped images of Fig. 8A [file 12885_2021_8915_MOESM18_ESM.pdf]

Supplementary Figure 17 (uncropped images of Figure 8B)

Dnmt3a

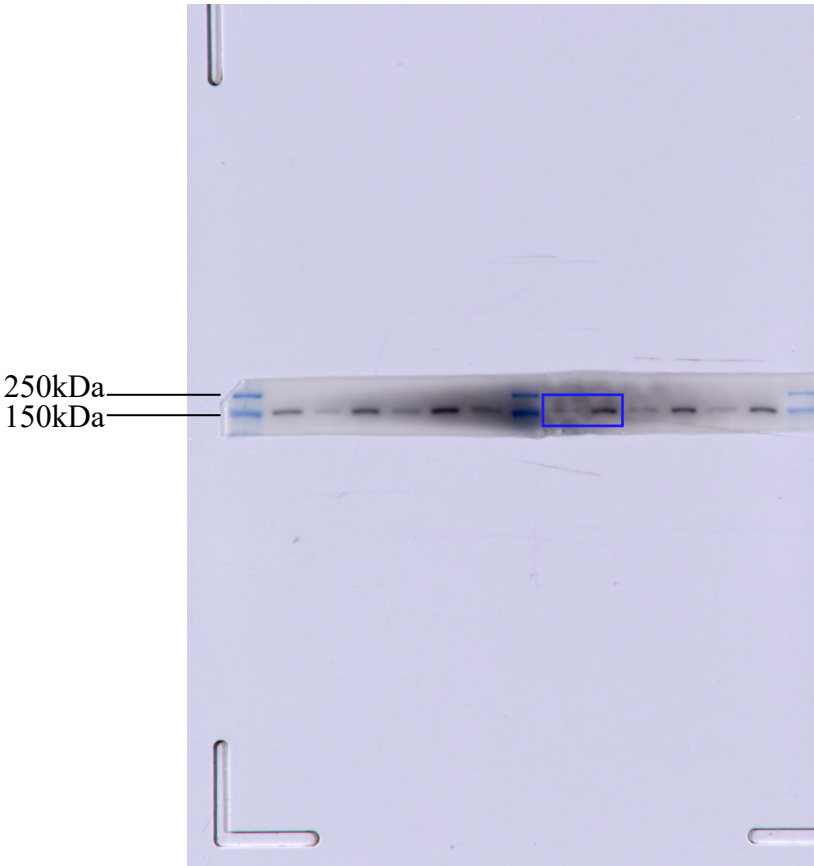

p-Stat5a

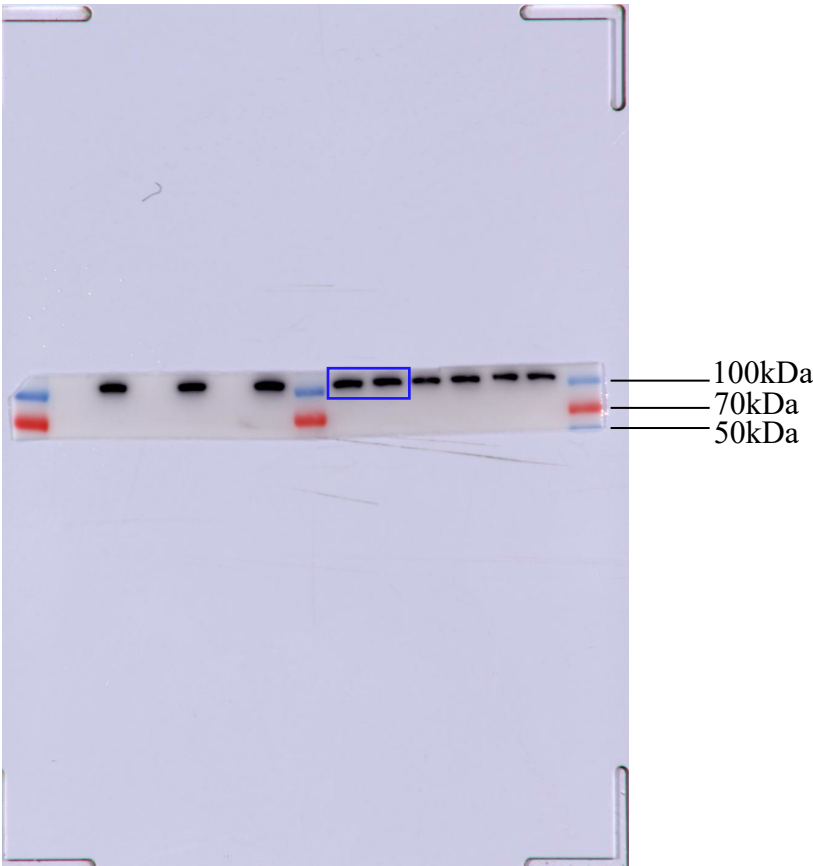

Cdkn1a

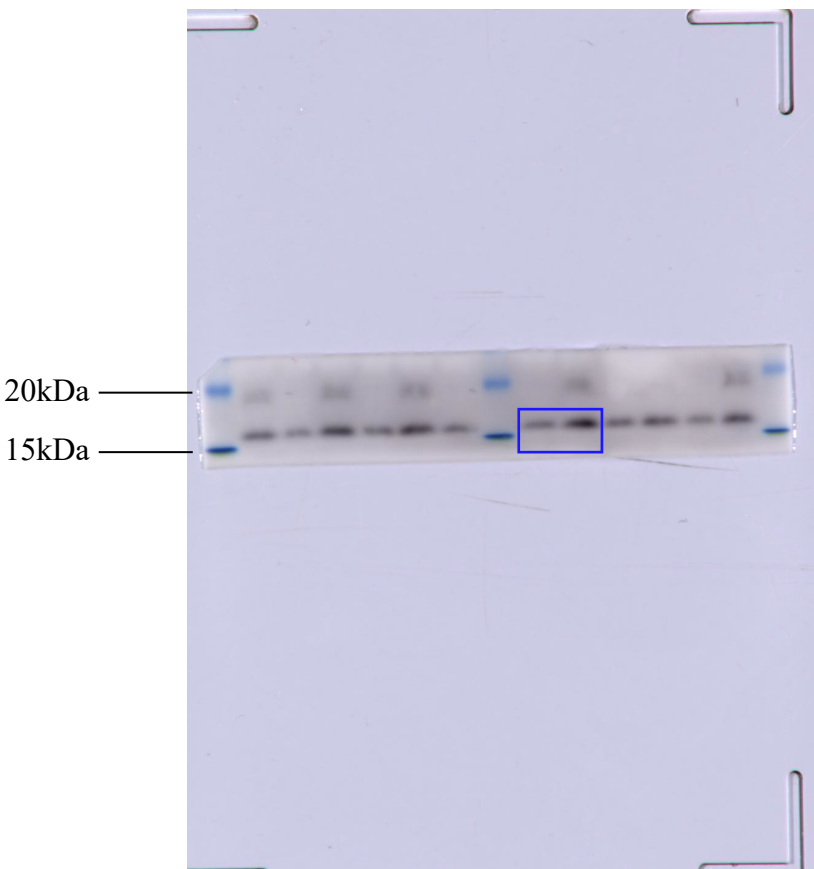

$\beta$ -actin

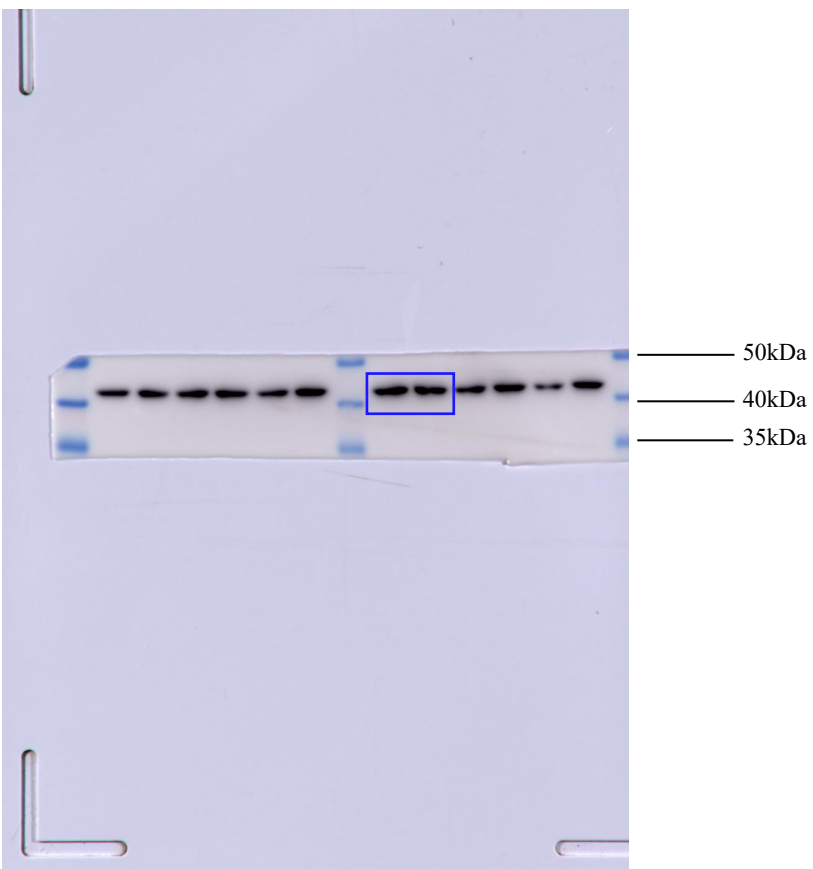

Supplement: Supplementary file 19 — Additional file 19: Fig. S17. Uncropped images of Fig. 8B [file 12885_2021_8915_MOESM19_ESM.pdf]

Supplementary Figure 18 (uncropped images of Figure 8C)

Dnmt3a

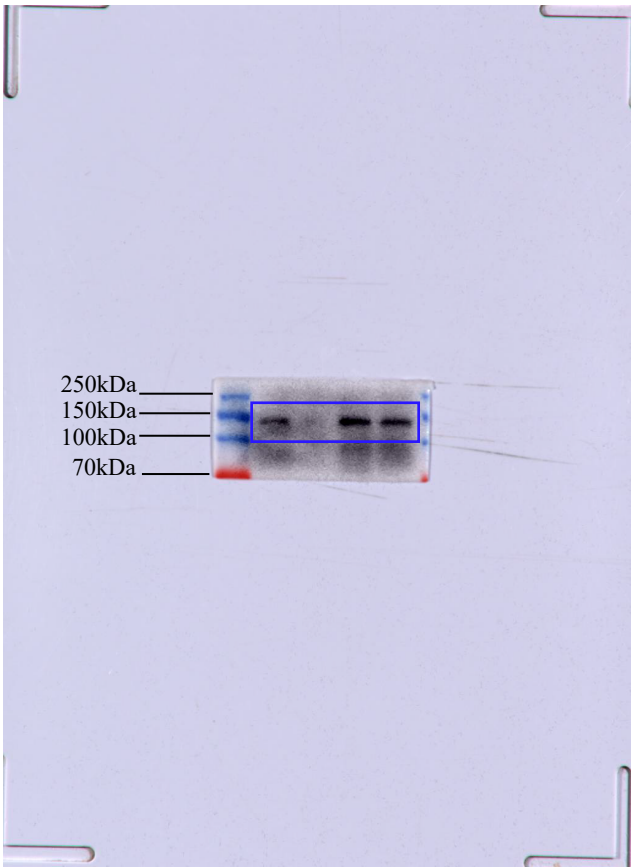

Cdkn1a

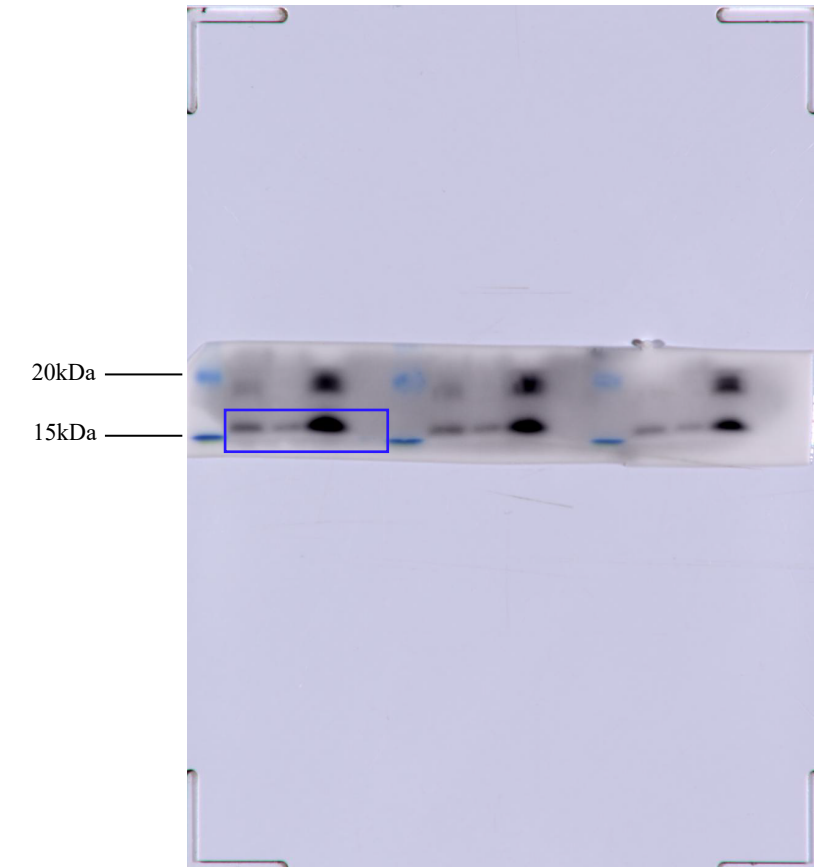

$\beta$ -actin

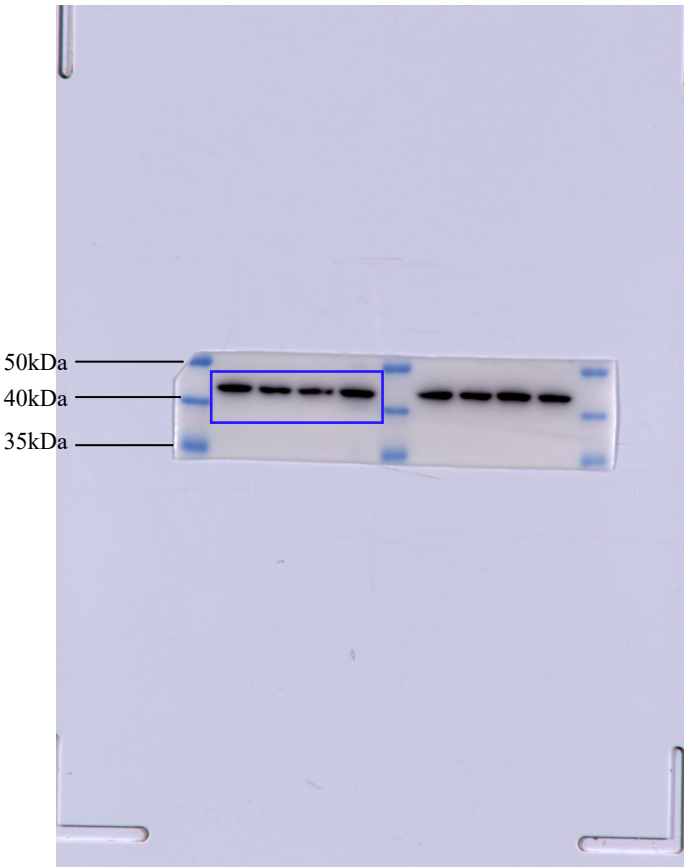

Supplement: Supplementary file 20 — Additional file 20: Fig. S18. Uncropped images of Fig. 8C [file 12885_2021_8915_MOESM20_ESM.pdf]
