# Supplementary material for: Dnmt3a is downregulated by Stat5a and mediates G0/G1 arrest by suppressing the miR-17-5p/Cdkn1a axis in Jak2V617F cells
Source: BMC Cancer. 2021 Nov 13;21:1213. doi: 10.1186/s12885-021-08915-0 (PMC8590245; doi:10.1186/s12885-021-08915-0)

Supplementary Figure 19 (uncropped images of Figure 9B and 9F)

Figure 9B

Dnmt3a

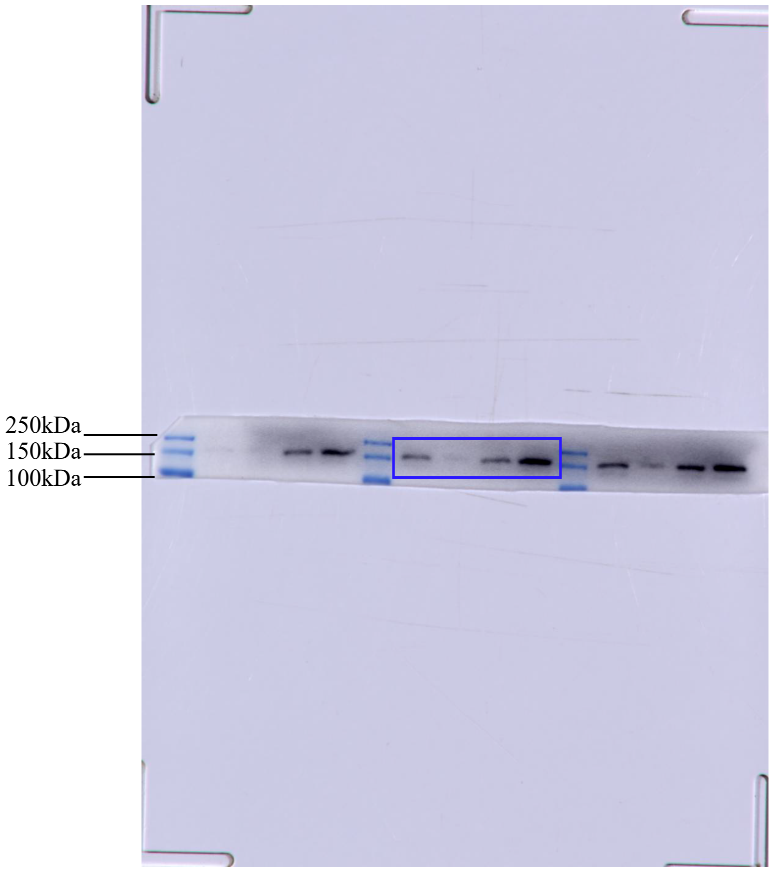

β-actin

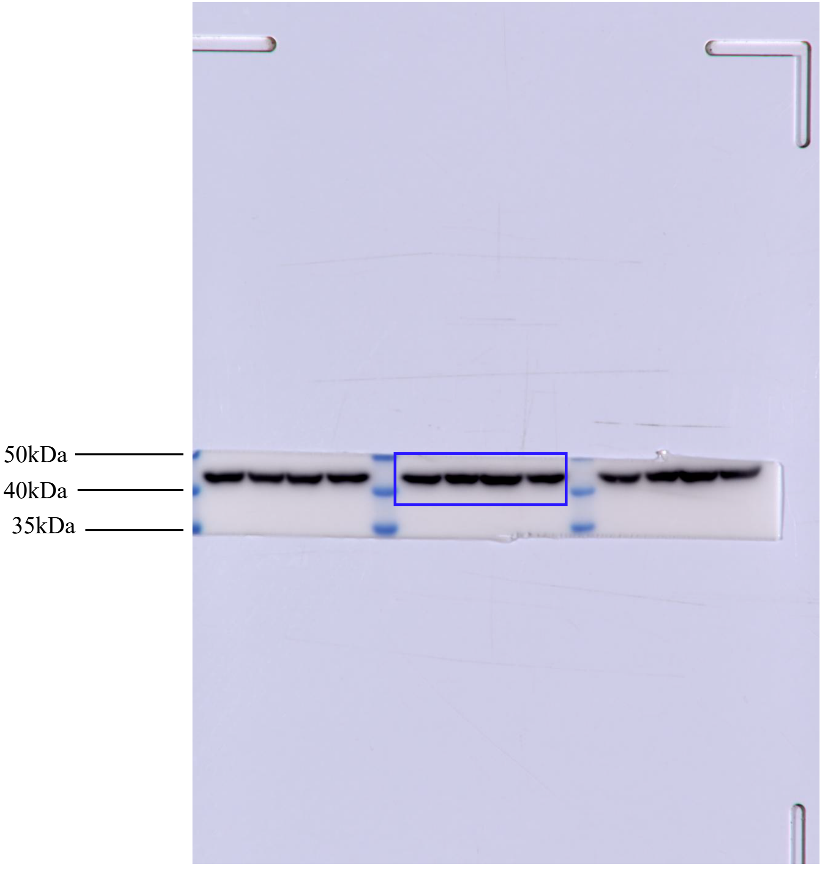

Figure 9F

Cdkn1a

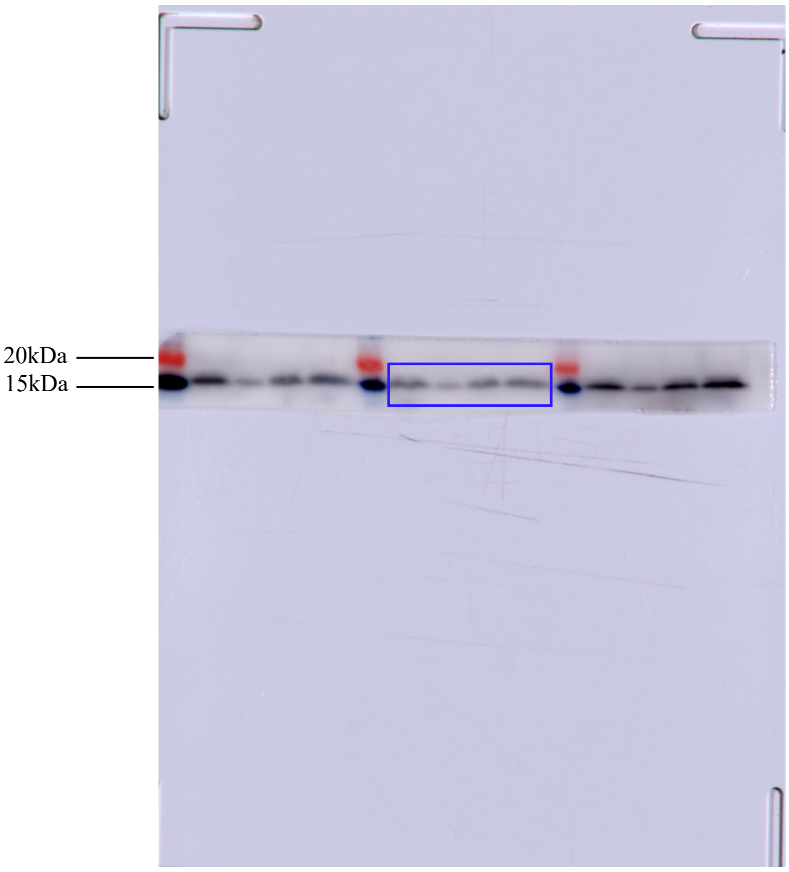

β-actin

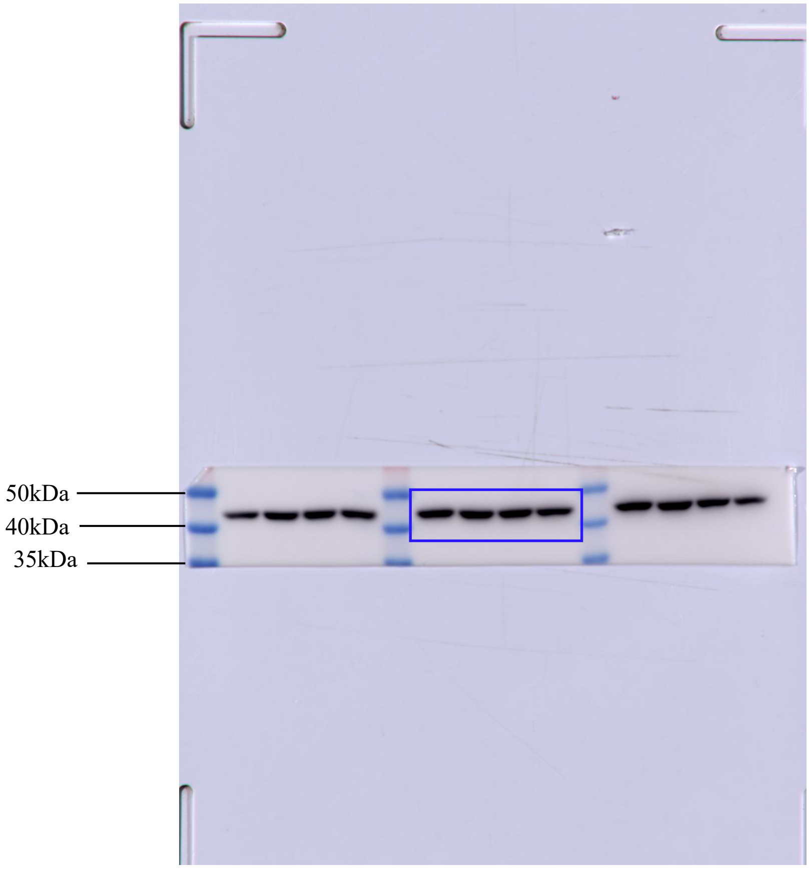

Supplement: Supplementary file 21 — Additional file 21: Fig. S19. Uncropped images of Fig. 9B and F [file 12885_2021_8915_MOESM21_ESM.pdf]
